# The Effect of Load and Volume Autoregulation on Muscular Strength and Hypertrophy: A Systematic Review and Meta-Analysis

**DOI:** 10.1186/s40798-021-00404-9

**Published:** 2022-01-15

**Authors:** Landyn M. Hickmott, Philip D. Chilibeck, Keely A. Shaw, Scotty J. Butcher

**Affiliations:** 1 College of Medicine, Health Sciences Program, University of Saskatchewan, Saskatoon, Canada; 2 College of Kinesiology, University of Saskatchewan, Saskatoon, Canada; 3 School of Rehabilitation Science, University of Saskatchewan, Saskatoon, Canada

## Abstract

**Background:**

Autoregulation has emerged as a potentially beneficial resistance training paradigm to individualize and optimize programming; however, compared to standardized prescription, the effects of autoregulated load and volume prescription on muscular strength and hypertrophy adaptations are unclear. Our objective was to compare the effect of autoregulated load prescription (repetitions in reserve-based rating of perceived exertion and velocity-based training) to standardized load prescription (percentage-based training) on chronic one-repetition maximum (1RM) strength and cross-sectional area (CSA) hypertrophy adaptations in resistance-trained individuals. We also aimed to investigate the effect of volume autoregulation with velocity loss thresholds ≤ 25% compared to > 25% on 1RM strength and CSA hypertrophy.

**Methods:**

This review was performed in accordance with the PRISMA guidelines. A systematic search of MEDLINE, Embase, Scopus, and SPORTDiscus was conducted. Mean differences (MD), 95% confidence intervals (CI), and standardized mean differences (SMD) were calculated. Sub-analyses were performed as applicable.

**Results:**

Fifteen studies were included in the meta-analysis: six studies on load autoregulation and nine studies on volume autoregulation. No significant differences between autoregulated and standardized load prescription were demonstrated for 1RM strength (MD = 2.07, 95% CI – 0.32 to 4.46 kg, *p* = 0.09, SMD = 0.21). Velocity loss thresholds ≤ 25% demonstrated significantly greater 1RM strength (MD = 2.32, 95% CI 0.33 to 4.31 kg, *p* = 0.02, SMD = 0.23) and significantly lower CSA hypertrophy (MD = 0.61, 95% CI 0.05 to 1.16 cm^2^, *p* = 0.03, SMD = 0.28) than velocity loss thresholds > 25%. No significant differences between velocity loss thresholds > 25% and 20–25% were demonstrated for hypertrophy (MD = 0.36, 95% CI – 0.29 to 1.00 cm^2^, *p* = 0.28, SMD = 0.13); however, velocity loss thresholds > 25% demonstrated significantly greater hypertrophy compared to thresholds ≤ 20% (MD = 0.64, 95% CI 0.07 to 1.20 cm^2^, *p* = 0.03, SMD = 0.34).

**Conclusions:**

Collectively, autoregulated and standardized load prescription produced similar improvements in strength. When sets and relative intensity were equated, velocity loss thresholds ≤ 25% were superior for promoting strength possibly by minimizing acute neuromuscular fatigue while maximizing chronic neuromuscular adaptations, whereas velocity loss thresholds > 20–25% were superior for promoting hypertrophy by accumulating greater relative volume.

*Protocol Registration* The original protocol was prospectively registered (CRD42021240506) with the PROSPERO (International Prospective Register of Systematic Reviews).

**Supplementary Information:**

The online version contains supplementary material available at 10.1186/s40798-021-00404-9.

## Key Points

- Autoregulated and standardized load prescription resulted in similar improvements in muscular strength. Subjective (repetitions in reserve-based rating of perceived exertion) and objective (velocity-based training) autoregulated load prescription also demonstrated similar improvements in muscular strength.
- Velocity loss thresholds ≤ 25% demonstrated significantly greater improvements in muscular strength compared to velocity loss thresholds > 25%; however, this was only evident when exercises in addition to the main resistance training intervention were performed. Velocity loss thresholds > 25% demonstrated significantly greater improvements in muscular hypertrophy compared to velocity loss thresholds ≤ 25%. No significant differences in hypertrophy were demonstrated between velocity loss thresholds of 20–25% and > 25%; however, velocity loss thresholds > 25% demonstrated significantly greater improvements in hypertrophy compared to velocity loss thresholds ≤ 20%.
- Future research is warranted investigating the chronic effects of autoregulated load and volume prescription with a more precise quantification of proximity to failure and in clinical populations when acute fluctuations in performance and chronic changes in adaptation are most apparent.

## Introduction

### Background

Resistance training (RT) is the principal modality to increase strength and hypertrophy for improving athletic performance and clinical health [1]. Traditionally, RT has been prescribed based on a pre-determined percentage of one-repetition maximum (1RM), which has been referred to in the scientific literature as standardized percentage-based training (PBT) [2]. There are, however, numerous limitations evident with PBT, the primary being that daily fluctuations [3] and short-term changes [4] in 1RM have been consistently observed [5]; therefore, PBT does not match the acute performance fluctuations and chronic physiological adaptations of each individual [6]. PBT also involves prescribing load based on a single 1RM testing session; thus, if abnormal performance or improper administration were present, the training stimulus applied for the study intervention or successive training cycle may be inappropriate for the intended outcome and may impact other variables (i.e., fatigue, load, volume) in the prescription [5]. Finally, repetitions performed at given intensities are largely lift-specific [7] and highly variable between individuals [8]; therefore, PBT fails to accurately quantify proximity to failure and the degree of neuromuscular fatigue for each individual and lift.

As an alternative approach to PBT, autoregulated RT has gained considerable popularity in recent years due to its theoretical ability to account for an individual’s changes in physiological adaptations and performance parameters [6]. Autoregulation may be defined as a two-step process of measurement and adjustment based on an individual’s acute and chronic fluctuations in performance (i.e., strength), in which performance is comprised of the sum of training (fitness and fatigue) and non-training (readiness) related factors [6]. The two predominant autoregulatory methods involve the systematic manipulation of load and volume via subjective and/or objective strategies [9]. Specifically, subjective load autoregulation involves implementing the repetitions in reserve-based rating of perceived exertion scale (RIR-based RPE scale) in an attempt to quantify proximity to failure, which is commonly referred to as RPE-based training [10]. However, due to the inaccuracy in intra-set RPE predictions [11], objective velocity-based training (VBT) [12] has emerged as a novel load autoregulatory strategy to rectify the limitations of subjective RPE-based training [13] and standardized PBT [14]. VBT load autoregulation involves either prescribing an average concentric velocity (ACV) zone corresponding to the force–velocity continuum [15, 16] or an individualized first repetition average concentric velocity (FRV) corresponding to a specific percentage of 1RM via an individualized load-velocity profile [17–19]. Despite the theoretical basis for autoregulated load prescription, the available evidence is conflicting whether it indeed provides an advantage over standardized load prescription for chronic muscular strength and hypertrophy adaptations. Although some studies have demonstrated that autoregulated load prescription may be superior to standardized load prescription for 1RM strength adaptations [15, 20] by enabling load to match the adaptation of the individual throughout a training study (i.e., enable higher relative intensities to be achieved) others have revealed no significant differences [19, 21–23].

Similar to subjective load autoregulation, subjective volume autoregulation involves implementing the RPE stop strategy, in which a particular number of sets are prescribed and each set is terminated at a pre-determined subjective RPE [24]. To date, no study has investigated the chronic effects of the RPE stop strategy on muscular strength and hypertrophy. Rather, objective velocity loss has emerged as the predominant strategy of volume autoregulation due to its inherent ability to accurately quantify acute intra-set neuromuscular fatigue [25]. Lower intra-set neuromuscular fatigue (i.e., lower velocity loss thresholds) may be superior for optimizing neuromuscular adaptations such as power output and shifts towards velocity-oriented force–velocity profiles; whereas higher intra-set neuromuscular fatigue (i.e., higher velocity loss thresholds) may be superior for optimizing muscular endurance [26]. The available evidence remains unclear which velocity loss thresholds optimize chronic strength and hypertrophy adaptations [27–36].

Proximity to failure and neuromuscular fatigue is of paramount importance when considering the design of RT programs [37]. Although training to failure has traditionally been promoted for overload [38, 39], this practice elevates muscle damage and elongates recovery time considerably compared to not training to failure [40]. Importantly, two recent systematic reviews and meta-analyses demonstrated no difference in hypertrophy between training to failure compared to not training to failure when volume was equalized [41, 42]. Similarly, two separate systematic reviews and meta-analyses also demonstrated no difference in hypertrophy between traditional sets compared to alternative set structures when relative intensity and relative volume were equated, which further demonstrates that considerable magnitudes of intra-set fatigue are unnecessary to promote hypertrophy [26, 37]. Despite this, all four systematic reviews and meta-analyses demonstrated no difference in strength adaptations between comparisons [26, 37, 41, 42]; however, appropriately managing the dynamic inter-play amidst proximity to failure and neuromuscular fatigue by integrating load and volume autoregulation strategies may have important practical implications [6, 43]. To illustrate, when sets and repetitions per set are matched, autoregulating set-to-set load to match the individual’s performance by training at closer proximities to failure (i.e., at higher relative intensities) results in significantly greater 1RM strength adaptations [20]. Alternatively, when sets and percentage of 1RM are matched, autoregulating intra-set volume with velocity loss thresholds (i.e., reducing neuromuscular fatigue) also results in significantly greater 1RM strength adaptations [28]. When equated for intra-set fatigue, the time course of recovery is similar regardless of the proximity to failure and relative intensity; however, when proximity to failure is equated, training with greater intra-set fatigue results in greater elevations in indirect measures of muscle damage compared to lower intra-set fatigue [44]. Crucially, excessive acute muscle damage attenuates high-threshold motor unit recruitment and motor skill learning; thus, impairing overall performance, training quality, and skill practice [45]. Chronic neuromuscular fatigue may reduce training frequency, an imperative variable implemented to increase volume and enhance hypertrophy to augment strength adaptations [46]. Overall, the potential practical implications but unclear efficacy of load and volume autoregulation justify the requirement to collate the existing literature and provide a comprehensive synthesis of the evidence.

### Objectives

The primary purpose of this review was to determine the chronic effects of load and volume autoregulation on 1RM strength adaptations, with cross-sectional area (CSA) muscle hypertrophy as a secondary outcome. Specifically, systematic and meta-analytic approaches were conducted to investigate the chronic effects of autoregulated compared to standardized load prescription and to investigate the chronic effects of autoregulated volume prescription via velocity loss thresholds ≤ 25% compared to velocity loss thresholds > 25%. It was hypothesized that autoregulated load prescription would result in significantly greater strength adaptations than standardized load prescription; however, no differences in hypertrophy would be observed. It was also hypothesized that velocity loss thresholds ≤ 25% would result in significantly greater strength adaptations, whereas velocity loss thresholds > 25% would result in significantly greater hypertrophy. Moreover, a quality assessment of the studies and limitations of the present autoregulatory methods were identified to suggest avenues for future research. The goal is that this review will provide comprehensive evidence regarding the efficacy of load and volume autoregulation for 1RM strength and CSA hypertrophy adaptations. Ultimately, the dissemination of this information may assist exercise professionals in the systematic individualization of resistance training programming.

## Methods

### Research Question

A systematic review and meta-analysis were performed in accordance with the Preferred Reporting Items for Systematic Reviews and Meta-Analyses (PRISMA) guidelines [47]. The original protocol was prospectively registered with the International Prospective Register of Systematic Reviews (PROSPERO) on April 8, 2021 (Registration number: CRD42021240506). A study intervention length of ≥ 5 weeks was selected because this corresponds to the approximate minimal amount of time required to observe significant hypertrophy with resistance training [48]. For volume autoregulation, a velocity loss threshold of ≤ 25% to > 25% was compared as ~ 20–30% velocity loss corresponds to ~ 50% of the maximal number of repetitions within a set [25] and ~ 25% velocity loss has typically been suggested to optimize 1RM strength adaptations [25, 30–32, 36]. The research question was defined using the participants, interventions, comparisons, outcomes, and study design (PICOS) framework:

1. *Participants:* Apparently healthy individuals with RT experience and no injury or health condition.
2. *Interventions:* RT interventions (≥ 5 weeks) that employed an autoregulated load prescription or volume autoregulation (≤ 25% velocity loss) protocol.
3. *Comparator:* RT interventions (≥ 5 weeks) that employed a standardized load prescription or volume autoregulation (> 25% velocity loss) protocol.
4. *Outcomes:* Muscular strength and/or muscular hypertrophy.
5. *Study design:* Prospective randomized or non-randomized comparative studies.

### Literature Search Strategy

A systematic search of the electronic databases MEDLINE, Embase, Scopus, and SPORTDiscus was performed to identify original research articles up to and including May 25, 2021. The searches had no language or date restrictions. The search strategy involved the following Boolean phrase of combined MeSH terms and keywords; ‘autoregulation’ OR ‘auto-regulation’ OR ‘load autoregulation’ OR ‘volume autoregulation’ OR ‘load prescription’ OR ‘volume prescription’ OR ‘rating of perceived exertion’ OR ‘repetitions in reserve’ OR ‘velocity-based training’ OR ‘velocity based training’ OR ‘velocity loss’ OR ‘absolute velocity’ OR ‘load-velocity profile’ OR ‘load velocity profile’ AND ‘powerlifting’ OR ‘power-lifting’ OR ‘power lifting’ OR ‘weightlifting’ OR ‘weight-lifting’ OR ‘weight lifting’ OR ‘weight-training’ OR ‘weight training’ OR ‘resistance-training’ OR ‘resistance training’ OR ‘resistance-exercise’ OR ‘resistance exercise’ OR ‘strength-training’ OR ‘strength training’ AND ‘one-repetition maximum’ OR ‘one repetition maximum’ OR ‘strength’ OR ‘musc* strength’ OR ‘hypertrophy’ OR ‘musc* hypertrophy’ OR ‘musc* size’ OR ‘musc* thickness’ OR ‘musc* cross-sectional area’.

### Study Selection

The Covidence systematic review software (Veritas Health Innovations, Melbourne, Australia) was used to screen titles and abstracts for full-text inclusion. Articles were deduplicated by Covidence and manually screened independently by LMH and KAS. Disagreements were resolved by consensus.

### Inclusion Criteria

The inclusion criteria for this review consisted of: (1) randomized or non-randomized comparative studies; (2) training intervention group that employed load or volume autoregulation; (3) strength and/or hypertrophy assessment pre- and post-intervention; (4) apparently healthy individuals with resistance training experience and no injury nor health condition; (5) ≥ 5 week resistance training intervention; (6) ≥ 2 times per week training frequency; (7) detailed description of training intervention including training intensity and training volume; (8) a validated device to measure and monitor velocity (for studies incorporating VBT). All grey literature (i.e., conferences, theses, reports, etc.) were excluded from the review.

### Data Extraction

The full texts of all articles that met the inclusion criteria for review were obtained for data extraction. The pre- and post-intervention data were extracted as mean differences (MD) ± standard deviations (SD). LMH extracted the relevant data of interest: (1) study information (study author and publication year); (2) participant characteristics (sample size, sex, age, height, weight, and training status); (3) training characteristics (prescription description, intervention length, training frequency, sets difference, repetitions difference, training volume, and training intensity). The authors of the selected articles were contacted to request any missing relevant information.

### Risk of Bias Assessment

The evaluation of risk of bias was performed using the Revised Cochrane risk-of-bias tool for randomized trials (RoB 2). LMH and KAS performed the methodological quality assessment independently. Disagreements were resolved by consensus.

### Statistical Analysis

All statistical analyses were conducted using RevMan (Version 5.3, Copenhagen, The Nordic Cochrane Centre, The Cochrane Collaboration). A fixed effects model was implemented to analyze the data. The data are reported as MD and 95% confidence intervals (CI). Standardized mean differences (SMD) were also determined to estimate effect sizes. An SMD of 0.20–0.49, 0.50–0.79, and ≥ 0.80 was considered a small, medium, and large effect, respectively. For load autoregulation with respect to 1RM strength outcomes, a positive and negative MD favored autoregulated and standardized load prescription, respectively. For volume autoregulation with respect to 1RM strength outcomes, a positive and negative MD favored velocity loss thresholds ≤ 25% and > 25%, respectively. For volume autoregulation with respect to CSA hypertrophy outcomes, a positive and negative MD favored velocity loss thresholds > 25% and ≤ 25%, respectively. Sub-analyses (i.e., intervention length, training frequency, etc.) and sub-group analyses (i.e., additional vs no additional exercise, etc.) were also performed. Results were considered statistically significant at *p* ≤ 0.05. The *I*^2^ and Chi^2^ statistics were used to assess heterogeneity. Funnel plots were used to assess publication bias.

## Results

### Study Selection

**Fig. 1:**
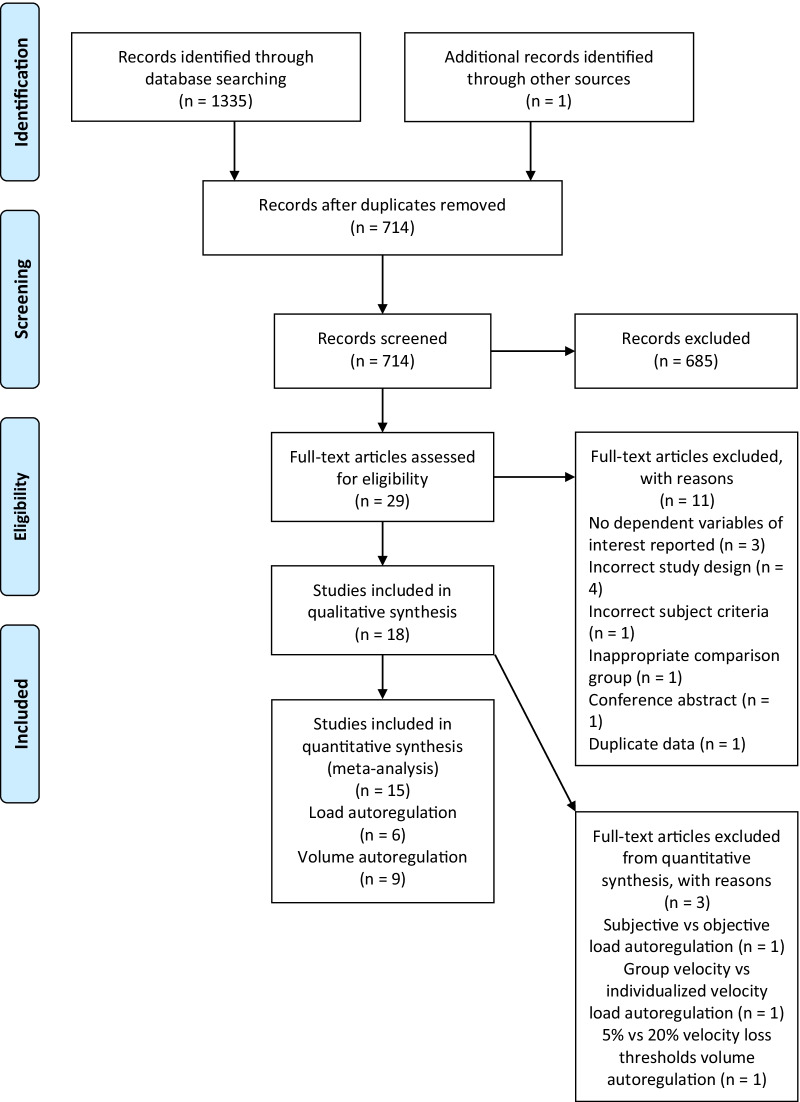
PRISMA (Preferred Reporting Items for Systematic Reviews and Meta-Analyses) flow diagram of literature search strategy. *n* number of studies

The PRISMA flow diagram outlining the literature search strategy is illustrated in Fig. 1. A total of 1336 studies were identified in the search, 18 of which were included in the systematic review: eight studies on load autoregulation and 10 studies on volume autoregulation. Of those 18 studies, 15 studies were included in the meta-analysis: six studies on load autoregulation and nine studies on volume autoregulation. Specifically, two studies on load autoregulation included in the systematic review were excluded from the meta-analysis; one study compared subjective load autoregulation via RPE-based training to objective load autoregulation via ACV zones [16], while the other study compared objective load autoregulation via group load-velocity profiles to objective load autoregulation via individualized load-velocity profiles [18]. One study on volume autoregulation included in the systematic review was excluded from the meta-analysis as the velocity loss thresholds in the two groups compared were both below 25% velocity loss (5% and 20% velocity loss) [27]. The authors of four studies were contacted to obtain relevant data of interest required to conduct the meta-analysis that were not presented in the published manuscripts. The author of two studies was contacted to obtain the pre- and post-test mean and SD for CSA of the vastus lateralis [31] and pectoralis major [32]. One author was contacted to verify the velocity loss threshold of the training to-repetition-failure group [28]. A final author was contacted to clarify the post-test mean and SD for 1RM of the bench press in the PBT group [21]. All authors responded and supplied the relevant data.

### Risk of Bias Assessment and Methodological Quality

Detailed summaries outlining the methodological quality of the included studies on autoregulated load and volume prescription are illustrated in Additional file 1: Table S1 and Additional file 2: Table S2, respectively. All studies had some risk of bias. Funnel plots for detecting publication bias of the included studies on load autoregulation for strength, volume autoregulation for strength, and volume autoregulation for hypertrophy are illustrated in Additional file 3: Fig. S1, Additional file 4: Fig. S2, and Additional file 5: Fig. S3, respectively. Visual inspection of the funnel plots indicated no obvious publication bias.

### Effect of Autoregulated Versus Standardized Load Prescription on Muscular Strength

#### Participant Characteristics

A detailed summary outlining the participant characteristics of the included studies on load autoregulation is illustrated in Table 1. A total of 133 participants (autoregulated: *n* = 64; standardized: *n* = 69) and 247 comparisons (autoregulated: *n* = 120; standardized: *n* = 127) were included in the meta-analysis. Five of the six studies included in the meta-analysis involved exclusively male participants aged 17 ± 1 to 28.3 ± 5.6 years old with ≥ 2 years of resistance-training experience [15, 19–22], and one study involved exclusively female participants aged 15.8 ± 1.3 years old with ≥ 1 year of resistance-training experience [23].

**Table 1:** Participant characteristics of included studies on load autoregulation

Study	Group	Number of participants	Sex distribution	Age (years)^a^	Height (cm)^a^	Weight (kg)^a^	Training status (subjective description; years of resistance-training experience)
Arede et al. [23]	PBT	7	F	15.8 ± 1.3	168.4 ± 4.5	60.2 ± 6.0	Resistance-trained female basketball players; ≥ 1 year
	RPE	7	F				
Banyard et al. [22]	PBT	12	M	26.2 ± 5.1	181.4 ± 7.4	84.2 ± 7.7	Resistance-trained males; ≥ 2 years
	VBT	12	M	25.5 ± 5.0	180.7 ± 8.5	84.7 ± 6.8	
Dorrell et al. [15]	PBT	8	M	22.8 ± 4.5	180.2 ± 6.4	89.3 ± 13.3	Resistance-trained males; ≥ 2 years
	VBT	8	M				
Dorrell et al. [18]	Group VBT	19	M	23.6 ± 3.7	182.7 ± 5.1	92.2 ± 8.7	Resistance-trained males; ≥ 2 years
	Individualized VBT		M				
Graham and Cleather [20]	PBT	16	M	28.3 ± 5.6	177.8 ± 6.5	82.5 ± 8.9	Resistance-trained males; ≥ 2 years
	RPE	15	M	27.9 ± 5.3	179.6 ± 6.5	83.2 ± 9.7	
Helms et al. [21]	PBT	11	M	23.8 ± 4.2	175 ± 8	80.2 ± 12.2	Resistance-trained males; ≥ 2 years
	RPE	10	M	20.9 ± 1.4	172 ± 6	78.8 ± 9.7	
Orange et al. [19]	PBT	15	M	17 ± 1	181 ± 6.3	84.9 ± 11.9	Resistance-trained male rugby players; ≥ 2 years
	VBT	12	M	17 ± 1	178 ± 5.3	81.8 ± 11.9	
Shattock and Tee [16]	Group 1	10	M	22 ± 3	NR	93.1 ± 14.5	Resistance-trained male rugby players; ≥ 2 years
	Group 2	10	M	23 ± 3	NR	95.6 ± 16.8	

*cm* centimetres, *F* female, *kg* kilograms, *M* male, *NR* not reported, *PBT* percentage-based training, *RPE* repetitions in reserve-based rating of perceived exertion training, *VBT* velocity-based training

^a^Data are presented as mean ± standard deviation

#### Training Characteristics

A detailed summary outlining the training characteristics of the included studies on load autoregulation is illustrated in Table 2. The length of the studies ranged from six to 12 weeks with a training frequency of two to three times per week. In all six studies that were included in the meta-analysis, the number of sets were matched, and in four studies, the repetitions were also matched. Outcome measures of interest for strength included 1RM of the back squat (six), bench press (three), deadlift (one), front squat (one), and overhead press (one). Outcome measures of interest for hypertrophy included muscle thickness of the vastus lateralis at 50% (one), vastus lateralis at 70% (one), and pectoralis major (one). A meta-analysis comparing the effect of autoregulated to standardized load prescription on muscular hypertrophy was unable to be conducted as only a single study measured and reported hypertrophy outcomes [21].

**Table 2:** Training characteristics of included studies on load autoregulation

Study	Group	Prescription	Length (weeks)	Frequency (days/week)	Sets difference	Repetitions difference	Average total volume difference	Average relative intensity difference	Outcomes of interest
Arede et al. [23]	PBT	Exercises: Back squat, bench press, hip thrust, shoulder press Week 1–4: 3 sets per exercise Week 5–8: 4 sets per exercise PBT group: 10 repetitions to failure RPE group: 7 repetitions to 7 RPE with set-to-set load adjustments of 2% increase/decrease per 0.5 RPE rating below/above RPE target	8	2	Matched	PBT: 2240 RPE: 1568	Greater for PBT	No significant difference	1RM back squat; 1RM bench press
	RPE								
Banyard et al. [22]	PBT	All sessions: 5 sets of 5 repetitions in back squat at loads ranging from ~ 59 to 85% of 1RM PBT group: Relative load based on pre-test 1RM VBT group: Sessional target velocity from individualized load-velocity profile and mean velocity table corresponding to identical relative load as PBT group with set-to-set load adjustments of 5% increase/decrease per 0.06 m^.^s^−1^ below/above the target velocity of all repetitions average velocity within set	6	3	Matched	Matched	No significant difference	No significant difference	1RM back squat
	VBT								
Dorrell et al. [15]	PBT	Integrated periodization structure comprised of compound exercises (back squat, bench press, deadlift, overhead press) and supplementary exercises PBT group: Relative load based on pre-test 1RM VBT group: Group velocity zones established from published data and pre-test 1RM corresponding to identical relative loads as PBT group with the integration of velocity stops at 20% below the target group velocity zone and set-to-set load adjustments if velocity outside target group velocity zone	6	2	Matched	Intended to be matched	Average relative volume load: Significantly greater in back squat for PBT (*p* = 0.033) Significantly greater in bench press for PBT (*p* = 0.019) No significant difference in deadlift (*p* = 0.398) Significantly greater in overhead press for PBT (*p* = 0.049)	NR	1RM back squat; 1RM bench press; 1RM deadlift; 1RM overhead press
	VBT								
Dorrell et al. [18]	Group VBT	Integrated periodization structure comprised of back squat and supplementary exercises Group VBT group: Group load-velocity profiles established from pre-test 1RM with target velocity corresponding to prescribed relative loads with the integration of velocity stops if a repetition velocity was below the target group velocity zone and set-to-set load adjustments based on group load-velocity profiles Individualized VBT group: Individualized load-velocity profiles established from pre-test 1RM with target velocity corresponding to prescribed relative loads with the integration of velocity stops if a repetition velocity was below the target individualized velocity zone and set-to-set load adjustments based on individualized load-velocity profiles	6	2	Matched	Intended to be matched	Average total volume load: No significant difference (*p* = 0.632)	NR	1RM back squat
	Individualized VBT								
Graham and Cleather [20]	PBT	Session 1: Front squat Session 2: Back squat Week 1–4: 3 sets of 10 repetitions per exercise Week 5–8: 4 sets of 5 repetitions per exercise Week 9–12: 3 sets of 3 repetitions per exercise PBT group: Relative load based on pre-test 1RM corresponding to 65, 67.5, 70, 72.5, 77.5, 80, 82.5, 85, 87.5, 90, 92.5, and 95% of 1RM for each single respective week from week 1–12 RPE group: Relative load intended to correspond similarly to PBT group and corresponding to 6, 7, 8, 9, 6, 7, 8, 9, 8, 9, 10, maximum RPE each single respective week from week 1–12	12	2	Matched	Matched	Average weekly volume load: No significant difference in combined back squat and front squat (*p* = 0.088)	Significantly greater in back squat for RPE (*p* = 0.006) Significantly greater in front squat for RPE (*p* < 0.001)	1RM back squat; 1RM front squat
	RPE								
Helms et al. [21]	PBT	Integrated periodization structure comprised of back squat and bench press Week 1 and 8: 2 sets per exercise Week 2–7: 3 sets per exercise PBT group: Relative load based on pre-test 1RM corresponding to 65–92.5% of 1RM RPE group: Relative load intended to correspond similarly to PBT group and corresponding to 5–10 RPE with set-to-set load adjustments of 2% increase/decrease per 0.5 RPE rating below/above RPE target	8	3	Matched	Matched	Average relative volume load: No significant difference in back squat (*p* = 0.66) Significantly greater in bench press for RPE (*p* = 0.03)	No significant difference in back squat (*p* = 0.49) Significantly greater in bench press for RPE (*p* < 0.001)	1RM back squat; 1RM bench press; MT vastus lateralis at 50%; MT vastus lateralis at 70%; MT pectoralis major
	RPE								
Orange et al. [19]	PBT	Exercises: Back squat and supplementary exercises Session 1: Back squat at 60% of 1RM Session 2: Back squat at 80% of 1RM PBT group: Relative load based on pre-test 1RM VBT group: Sessional target velocity from individualized load-velocity profile corresponding to identical relative load as PBT group with set-to-set load adjustments of 5% increase/decrease per 0.06 m^.^s^−1^ below/above target velocity of first repetition velocity within set	7	2	Matched	Matched	No significant difference	No significant difference	1RM back squat
	VBT								
Shattock and Tee [16]	Group 1	Comprised of compound exercises and supplementary exercises Week 1–6: Maximal-strength block Week 7–12: Strength-speed block VBT prescription: Group velocity zones established from published data with target velocity corresponding to prescribed relative loads with set-to-set load adjustments if velocity outside target group velocity zone RPE prescription: Relative load intended to correspond similarly to VBT prescription with load dictated via RPE and set-to-set load adjustments if RPE outside target RPE zone	12	Week 1–6 = 4 Week 7–12 = 3	Matched	Matched	No significant difference	NR	1RM back squat; 1RM bench press
	Group 2								

*MT* muscle thickness, *m*^*.*^s^−1^ m per second, *NR* not reported, *PBT* percentage-based training, *RPE* repetitions in reserve-based rating of perceived exertion training, *VBT* velocity-based training, *1RM* one-repetition maximum

#### Strength Outcomes

**Fig. 2:**
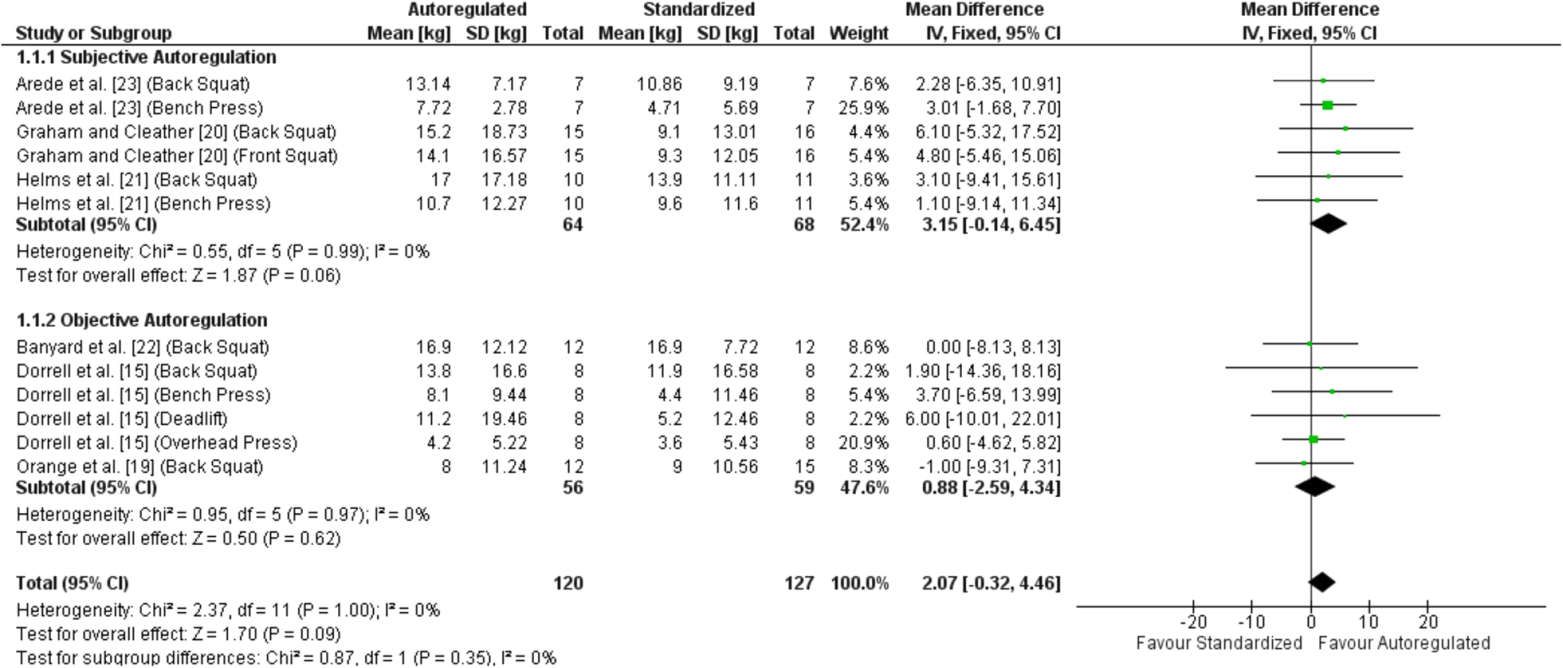
Forest plot for fixed effects meta-analysis of the mean differences in one-repetition maximum strength adaptations comparing autoregulated to standardized load prescription with subgroup analysis comparing subjective to objective autoregulation. *CI* confidence interval, *df* degrees of freedom, *kg* kilograms, *SD* standard deviation

Five of the six studies measured muscular strength with a 1RM test [15, 19–22], while the remaining study estimated 1RM in accordance with the Brzycki [49] prediction equation using a 10RM test [23]. The overall pooled analysis revealed no significant difference for 1RM strength adaptations between autoregulated and standardized load prescription (MD = 2.07, 95% CI – 0.32 to 4.46 kg, *p* = 0.09, SMD = 0.21) as illustrated in Fig. 2. Specifically, three studies employed subjective load autoregulation [20, 21, 23] and three studies employed objective load autoregulation [15, 19, 22], comprising a total of six outcome measures for each autoregulation type. The sub-analysis revealed near-significance with a small effect favoring autoregulated over standardized load prescription when subjective autoregulation was employed (MD = 3.15, 95% CI – 0.14 to 6.45 kg, *p* = 0.06, SMD = 0.30), but no meaningful difference when objective autoregulation was employed (MD = 0.88, 95% CI – 2.59 to 4.34 kg, *p* = 0.62, SMD = 0.10). Despite this finding, the pooled subgroup analysis comparing subjective to objective load autoregulation revealed no significant difference (*p* = 0.35).

Sub-analyses are presented in Additional file 6: Table S3. The sub-analysis revealed near-significance with a small effect favoring autoregulated over standardized load prescription when the training intervention was ≥ 8 weeks (*p* = 0.06, SMD = 0.30), but no significant differences for all other sub-analyses (i.e., when the training intervention was < 8 weeks, when the training frequency was 3 times per week or < 3 times per week, when training volume was controlled or uncontrolled, when relative intensity was greater for autoregulated or the same as standardized load prescription, when lower or upper body exercises were assessed separately, when squat or bench press were assessed separately, or when exercises additional to the resistance training were performed or not performed).

### Effect of Volume Autoregulation on Muscular Strength and Hypertrophy

#### Participant Characteristics

A detailed summary outlining the participant characteristics of the included studies on volume autoregulation is illustrated in Table 3. A total of 308 participants (≤ 25% velocity loss: *n* = 171; > 25% velocity loss: *n* = 137) and 457 comparisons (≤ 25% velocity loss: *n* = 230; > 25% velocity loss: *n* = 227) were included in the meta-analysis. All studies involved male participants, while one study also involved four female participants. All participants possessed resistance-training experience and the age ranged from 19.4 ± 1.7 to 26.7 ± 5.5 years old.

**Table 3:** Participant characteristics of included studies on volume autoregulation

Study	Group	Number of participants	Sex distribution	Age (years)^a^	Height (cm)^a^	Weight (kg)^a^	Training status (subjective description; years of resistance-training experience)
Galiano et al. [27]	VL5	15	M	22.1 ± 2.9	175.1 ± 5.3	72.5 ± 11.3	Resistance-trained males; ≥ 1.5 years
	VL20	13	M	23.9 ± 3.0	176.6 ± 3.5	75.7 ± 9.4	
Held et al. [28]	VL10	11	9 M/2 F	19.8 ± 2.3	184 ± 5	75.8 ± 8.6	Resistance-trained male and female rowers; ≥ 2 years
	TRF	10	8 M/2 F	19.4 ± 1.7	180 ± 10	73.3 ± 8.9	
Pareja-Blanco et al. [29]	VL15	8	M	23.8 ± 3.4	174 ± 7	75.5 ± 8.6	Resistance-trained professional male soccer players; NR
	VL30	8	M				
Pareja-Blanco et al. [30]	VL20	12	M	22.7 ± 1.9	176 ± 6	75.8 ± 7.0	Resistance-trained males; ≥ 1.5 years
	VL40	10	M				
Pareja-Blanco et al. [31]	VL0	14	M	24.1 ± 4.3	175 ± 5.5	75.5 ± 9.7	Resistance-trained males; ≥ 1.5 years
	VL10	14	M				
	VL20	13	M				
	VL40	14	M				
Pareja-Blanco et al. [32]	VL0	15	M	24.1 ± 4.3	175 ± 5.5	75.5 ± 9.7	Resistance-trained males; ≥ 1.5 years
	VL15	16	M				
	VL25	15	M				
	VL50	16	M				
Rodiles-Guerrero et al. [33]	VL10	15	M	23.0 ± 2.0	173 ± 5	73.3 ± 5.9	Resistance-trained males; ≥ 1 year
	VL30	15	M				
	VL50	15	M				
Rodríguez-Rosell et al. [34]	VL10	12	M	22.8 ± 3.1	177 ± 8	75.1 ± 10.3	Resistance-trained males; ≥ 1 year
	VL30	13	M	22.2 ± 2.7	176 ± 7	74.0 ± 9.1	
Rodríguez-Rosell et al. [35]	VL10	11	M	22.8 ± 3.9	176 ± 4	70.7 ± 5.1	Resistance-trained males; ≥ 1 year
	VL30	11	M	21.9 ± 2.3	176 ± 7	73.7 ± 9.4	
	VL45	11	M	21.6 ± 2.8	172 ± 8	72.1 ± 9.6	
Sánchez-Moreno et al. [36]	VL25	15	M	26.7 ± 5.5	175.8 ± 6	74.1 ± 4.7	Resistance-trained males; ≥ 2 years
	VL50	14	M	24.8 ± 6.1	176.1 ± 5	74.3 ± 8.1	

^a^Data are presented as mean ± standard deviation

*cm* centimetres, *F* female, *kg* kilograms, *M* male, *NR* not reported, *TRF* to-repetition-failure, *VL* percentage velocity loss

#### Training Characteristics

A detailed summary outlining the training characteristics of the included studies on volume autoregulation is illustrated in Table 4. The length of the studies ranged from five to eight weeks with a training frequency of two to three times per week. In all nine studies, the number of sets were matched; however, the total number of repetitions performed varied and increased concomitantly with an increasing velocity loss threshold employed. Outcome measures of interest for strength included 1RM of the back squat (one), bench press (one), deadlift (one), bench row (one), smith machine back squat (five), smith machine bench press (one), weight stack bench press (one), and body mass prone-grip pullup (one). Outcome measures of interest for hypertrophy included CSA of the rectus femoris (one), vastus lateralis (one), vastus lateralis + vastus intermedius (one), vastus medialis (one), and pectoralis major (one).

**Table 4:** Training characteristics of included studies on volume autoregulation

Study	Group	Prescription	Length (weeks)	Frequency (days/week)	Sets difference	Repetitions difference^a^	Average relative volume difference	Average relative intensity difference	Outcomes of interest
Galiano et al. [27]	VL5	Smith machine back squat, 3 sets per session, each group terminated each set at respective VL threshold, 3 min inter-set rest, absolute loads adjusted for each session from the first repetition of the first set to ensure velocity (± 0.03 m^.^s^−1^) matched prescribed % of 1RM (based on 50% of 1RM corresponding to 1.14 m^.^s^−1^) Session 1–14: 50% of 1RM	7	2	Matched	VL5: 156.9 ± 25.0 VL20: 480.5 ± 162.0	Significantly greater for VL20	No significant difference	1RM smith machine back squat
	VL20								
Held et al. [28]	VL10	Power clean + back squat + bench row + deadlift + bench press, 4 sets per exercise per session, VL10 group terminated each set at VL10 threshold and TRF group performed each set to- repetition-failure, 2–3 min inter-set rest Session 1–16: 80% of 1RM Day 1: 40 min of supplementary training Day 2: Resistance training, 90 min of low-intensity rowing Day 3: 90 min of low-intensity rowing, 60 min of optional low-intensity cross-training (running and biking) Day 4: 120 min of low-intensity cross-training (running and biking) Day 5: Resistance training, 60 min of low-intensity cross-training (running and biking) Day 6: 90 min of low-intensity rowing, 120 min of optional low-intensity cross-training (running and biking) Day 7: 3 sets of 2000 m high-intensity rowing	8	2	Matched	VL10: 2145 ± 285 TRF: 2825 ± 100	Significantly greater for TRF	No significant difference	1RM back squat; 1RM bench press; 1RM deadlift; 1RM bench row
	TRF								
Pareja-Blanco et al. [29]	VL15	Smith machine back squat, 2–3 sets per session, each group terminated each set at respective VL threshold, 4 min inter-set rest, absolute loads adjusted for each session from the first repetition of the first set to ensure velocity (± 0.03 m^.^s^−1^) matched prescribed % of 1RM (based on 50% of 1RM corresponding to 1.13 m^.^s^−1^, 55% of 1RM to 1.06 m^.^s^−1^, 60% of 1RM to 0.98 m^.^s^−1^, 65% of 1RM to 0.90 m^.^s^−1^, and 70% of 1RM to 0.82 m^.^s^−1^) Session 1–3: 50% of 1RM Session 4–6: 55% of 1RM Session 7–10: 60% of 1RM Session 11–14: 65% of 1RM Session 15–17: 70% of 1RM Session 18: 60% of 1RM	6	3	Matched	VL15: 251.2 ± 55.4 VL30: 414.6 ± 124.9	Significantly greater for VL30	No significant difference	1RM smith machine back squat
	VL30								
Pareja-Blanco et al. [30]	VL20	Smith machine back squat, 3 sets per session, each group terminated each set at respective VL threshold, 4 min inter-set rest, absolute loads adjusted for each session from the first repetition of the first set to ensure velocity (± 0.03 m^.^s^−1^) matched prescribed % of 1RM (based on 70% of 1RM corresponding to 0.82 m^.^s^−1^, 75% of 1RM to 0.75 m^.^s^−1^, 80% of 1RM to 0.68 m^.^s^−1^, and 85% of 1RM to 0.60 m^.^s^−1^) Session 1–6: 70% of 1RM Session 7–10: 75% of 1RM Session 11–13: 80% of 1RM Session 14–16: 85% of 1RM	8	2	Matched	VL20: 185.9 ± 22.2 VL40: 310.5 ± 42.0	Significantly greater for VL40	No significant difference	1RM smith machine back squat; CSA rectus femoris; CSA vastus lateralis + vastus intermedius; CSA vastus medialis
	VL40								
Pareja-Blanco et al. [31]	VL0	Smith machine back squat, 3 sets per session, each group terminated each set at respective VL threshold, 4 min inter-set rest, absolute loads adjusted for each session from the first repetition of the first set to ensure velocity (± 0.03 m^.^s^−1^) matched prescribed % of 1RM (based on individualized load-velocity profile) Session 1–5: 70% of 1RM Session 6–10: 75% of 1RM Session 11–14: 80% of 1RM Session 15–16: 85% of 1RM	8	2	Matched	VL0: 48.0 ± 0.0 VL10: 143.6 ± 40.2 VL20: 168.5 ± 47.4 VL40: 305.6 ± 81.7	VL40 significantly greater than VL0, VL10, and VL20; VL10 and VL20 significantly greater than VL0; No significant difference between VL10 and VL20	No significant difference	1RM smith machine back squat; CSA vastus lateralis
	VL10								
	VL20								
	VL40								
Pareja-Blanco et al. [32]	VL0	Smith machine bench press, 3 sets per session, each group terminated each set at respective VL threshold, 4 min inter-set rest, absolute loads adjusted for each session from the first repetition of the first set to ensure velocity (± 0.03 m^.^s^−1^) matched prescribed % of 1RM (based on individualized load-velocity profile) Session 1–5: 70% of 1RM Session 6–10: 75% of 1RM Session 11–14: 80% of 1RM Session 15–16: 85% of 1RM	8	2	Matched	VL0: 48.0 ± 0.0 VL15: 136.6 ± 17.8 VL25: 191.1 ± 34.1 VL50: 316.4 ± 65.1	VL50 significantly greater than VL0, VL15, and VL25; VL15 and VL25 significantly greater than VL0; No significant difference between VL15 and VL25	No significant difference	1RM smith machine bench press; CSA pectoralis major
	VL15								
	VL25								
	VL50								
Rodiles-Guerrero et al. [33]	VL10	Weight stack bench press, 4 sets per session, each group terminated each set at respective VL threshold, 3 min inter-set rest, absolute loads adjusted for each session from the first repetition of the first set to ensure velocity (± 0.03 m^.^s^−1^) matched prescribed % of 1RM (based on 65% of 1RM corresponding to 0.67 m^.^s^−1^, 70% of 1RM to 0.60 m^.^s^−1^, 75% of 1RM to 0.53 m^.^s^−1^, 80% of 1RM to 0.46 m^.^s^−1^, and 85% of 1RM to 0.39 m^.^s^−1^) Session 1–3: 65% of 1RM Session 4–6: 70% of 1RM Session 7–9: 75% of 1RM Session 10–12: 80% of 1RM Session 13–15: 85% of 1RM	5	3	Matched	VL10: 211.1 ± 17.3 VL30: 398.1 ± 61.4 VL50: 444.4 ± 51.9	VL50 significantly greater than VL30 and VL10; VL30 significantly greater than VL10	No significant difference	1RM weight stack bench press
	VL30								
	VL50								
Rodríguez-Rosell et al. [34]	VL10	Smith machine back squat, 3 sets per session, each group terminated each set at respective VL threshold, 4 min inter-set rest, absolute loads adjusted for each session from the first repetition of the first set to ensure velocity (± 0.03 m^.^s^−1^) matched prescribed % of 1RM (based on 70% of 1RM corresponding to 0.84 m^.^s^−1^, 75% of 1RM to 0.75 m^.^s^−1^, 80% of 1RM to 0.68 m^.^s^−1^, and 85% of 1RM to 0.60 m^.^s^−1^) Session 1–6: 70% of 1RM Session 7–10: 75% of 1RM Session 11–13: 80% of 1RM Session 14–16: 85% of 1RM	8	2	Matched	VL10: 109.6 ± 2.0 VL30: 228.0 ± 76.6	VL30 significantly greater than VL10	No significant difference	1RM smith machine back squat
	VL30								
Rodríguez-Rosell et al. [35]	VL10	Smith machine back squat, 3 sets per session, each group terminated each set at respective VL threshold, 4 min inter-set rest, absolute loads adjusted for each session from the first repetition of the first set to ensure velocity (± 0.03 m^.^s^−1^) matched prescribed % of 1RM (based on 55% of 1RM corresponding to 1.08 m^.^s^−1^, 60% of 1RM to 1.00 m^.^s^−1^, 65% of 1RM to 0.92 m^.^s^−1^, and 70% of 1RM to 0.84 m^.^s^−1^) Session 1–5: 55% of 1RM Session 6–9: 60% of 1RM Session 10–13: 65% of 1RM Session 14–16: 70% of 1RM	8	2	Matched	VL10: 180.8 ± 29.0 VL30: 347.9 ± 62.3 VL45: 501.1 ± 106.8	VL45 significantly greater than VL30 and VL10; VL30 significantly greater than VL10	No significant difference	1RM smith machine back squat
	VL30								
	VL45								
Sánchez-Moreno et al. [36]	VL25	Body mass prone-grip pullup, each group terminated each set at respective VL threshold, 3-min inter-set rest Session 1–3: 2 sets Session 4–8: 3 sets Session 9–14: 4 sets Session 15: 3 sets Session 16: 2 sets	8	2	Matched	VL25: 363.0 ± 84.6 VL50: 556.3 ± 121.9	VL50 significantly greater than VL25	No significant difference	1RM body mass prone-grip pullup
	VL50								

^a^Data are presented as mean ± standard deviation

*CSA* cross-sectional area, *m*^*.*^*s*^*−1*^ m per second, *TRF* to-repetition-failure, *VL* percentage velocity loss, *1RM* one-repetition maximum

#### Strength Outcomes

**Fig. 3:**
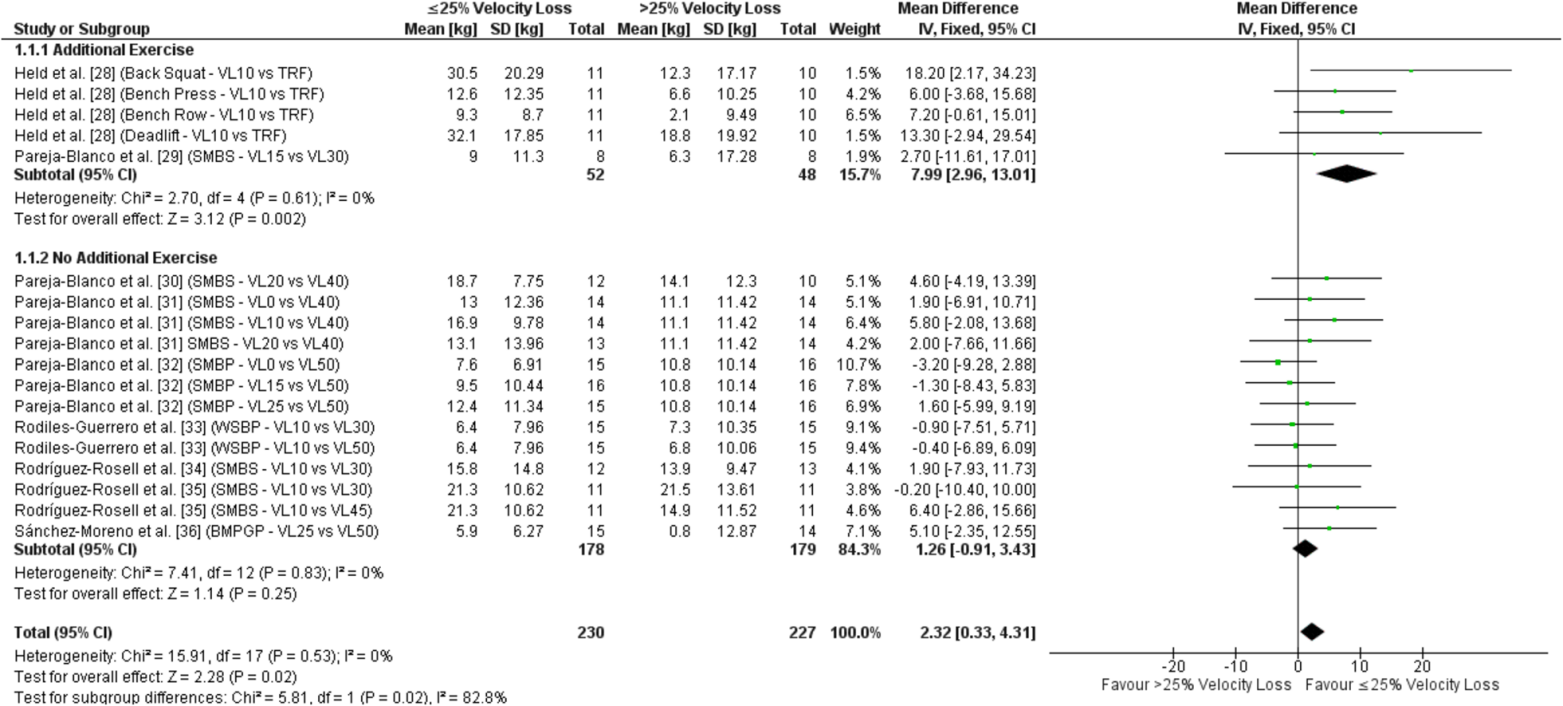
Forest plot for fixed effects meta-analysis of the mean differences in one-repetition maximum strength adaptations comparing ≤ 25% to > 25% velocity loss with subgroup analysis comparing additional to no additional exercise apart from the main comparator resistance training protocol. *BMPGP* body mass prone-grip pullup, *CI* confidence interval, *df* degrees of freedom, *kg* kilograms, *SD* standard deviation, *SMBP* smith machine bench press, *SMBS* smith machine back squat, *TRF* to-repetition-failure, *VL* percentage velocity loss, *WSBP* weight stack bench press

All nine studies reported our primary outcome measure of muscular strength and employed a 1RM test [28–36]. In studies comparing ≥ 3 velocity loss threshold groups, the velocity loss thresholds were separated into their respective groups for comparisons (i.e., ≤ 25% or > 25% velocity loss). Therefore, our overall meta-analysis for strength was comprised of 230 and 227 comparisons for velocity loss thresholds ≤ 25% and > 25%, respectively. The overall pooled analysis revealed a significant difference for 1RM strength adaptations favoring velocity loss thresholds ≤ 25% over velocity loss thresholds > 25% (Fig. 3; *p* = 0.02, SMD = 0.23). The sub-analysis revealed a significant difference for 1RM strength adaptations favoring velocity loss thresholds ≤ 25% over velocity loss thresholds > 25% when exercise in addition to the resistance training protocol was performed (*p* = 0.002, SMD = 0.62), but not for when no additional exercise was performed (*p* = 0.25, SMD = 0.13). Importantly, the pooled subgroup analysis comparing additional exercise to no additional exercise also revealed a significant difference (*p* = 0.02).

Sub-analyses are presented in Additional file 7: Table S4. The sub-analysis revealed a significant difference for 1RM strength favoring velocity loss thresholds ≤ 25% over velocity loss thresholds > 25% when the training intervention was 8 weeks (*p* = 0.009), when training frequency was < 3 times per week (*p* = 0.009), in lower body exercises (*p* = 0.007), in the smith machine back squat (*p* = 0.05), and in free-weight exercises (*p* = 0.0007), but no meaningful difference when the training intervention was < 8 weeks, when training frequency was 3 times per week, in upper body exercises, in the smith machine / weight stack bench press, or in machine-based exercises. The results from all additional sub-analyses for 1RM strength adaptations comparing each velocity loss threshold and every range iteration from 0–25% compared to > 25% are presented in Additional file 8: Table S5. Significantly greater 1RM strength adaptations were demonstrated for velocity loss thresholds of 10%, 10–25%, 10–20%, 0–20%, and 10–15% compared to velocity loss thresholds > 25%.

#### Hypertrophy Outcomes

**Fig. 4:**
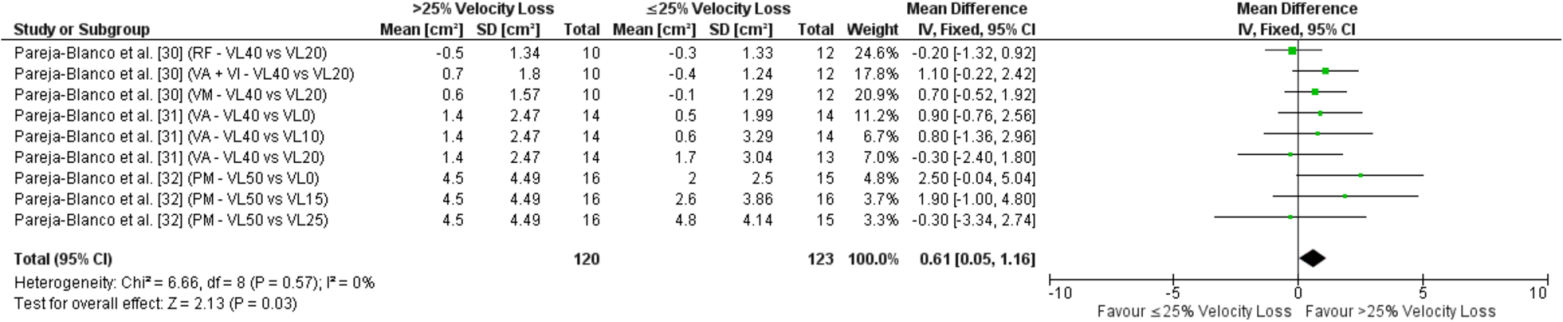
Forest plot for fixed effects meta-analysis of the mean differences in cross-sectional area hypertrophy adaptations comparing > 25% to ≤ 25% velocity loss. *CI* confidence interval, *cm* centimetres, *df* degrees of freedom, *PM* pectoralis major, *RF* rectus femoris, *SD* standard deviation, *VA* vastus lateralis, *VI* vastus intermedius, *VL* percentage velocity loss, *VM* vastus medialis

A total of three studies [30–32] reported our secondary outcome measure of muscular hypertrophy measured as muscle CSA. Similar to strength outcomes, in studies comparing ≥ 3 velocity loss threshold groups, the velocity loss thresholds were separated into their respective groups for comparisons (i.e., > 25% or ≤ 25% velocity loss). Therefore, our overall meta-analysis for hypertrophy was comprised of 120 and 123 comparisons for velocity loss thresholds > 25% and ≤ 25%, respectively. The overall pooled analysis revealed a significant difference for CSA hypertrophy adaptations favoring velocity loss thresholds > 25% over velocity loss thresholds ≤ 25% (MD = 0.61, 95% CI 0.05 to 1.16 cm^2^, *p* = 0.03, SMD = 0.28) as illustrated in Fig. 4. The results from all sub-analyses for CSA hypertrophy adaptations comparing velocity loss thresholds > 25% to each velocity loss threshold and every range iteration from 0–25% are presented in Additional file 9: Table S6. The sub-analysis revealed no significant difference and negligible effects for CSA hypertrophy when comparing velocity loss thresholds > 25% to velocity loss thresholds of 20–25%; however, all additional sub-analyses revealed small-to-moderate effects in favor of > 25%. Significantly greater CSA hypertrophy adaptations were demonstrated for velocity loss thresholds > 25% compared to velocity loss thresholds of 0–20%, 0–15%, 0–10%, and 0%.

## Discussion

To the authors’ knowledge, this is the first systematic review and meta-analysis to directly investigate the effect of load and volume autoregulation on muscular strength and hypertrophy adaptations. There were similar improvements in 1RM strength between autoregulated and standardized load prescription. Moreover, the subgroup analysis demonstrated no difference in 1RM strength whether subjective or objective autoregulated load prescription was employed. For volume autoregulation, low-moderate velocity loss thresholds (≤ 25%) were optimal for 1RM strength, which was only apparent when studies included exercise outside of the main comparator training protocol. Conversely, moderate-high velocity loss thresholds (> 25%) were optimal for muscle hypertrophy; however, velocity loss thresholds of 20 and 25% stimulate similar improvements in hypertrophy. Collectively, velocity loss thresholds of ~ 20–25% may be optimal for 1RM strength adaptations plausibly by promoting type II phenotype muscle hypertrophy [30], and potentially by maximizing chronic neuromuscular adaptations (i.e., late rate of force development) [32] while minimizing excessive and unnecessary acute neuromuscular fatigue [44]. Appropriate prescription of load and volume autoregulation strategies may enable the stimulus to parallel an individual’s performance and overarching goals of a resistance training program.

### Load Autoregulation

#### Muscular Strength

Overall, there was no significant difference in 1RM strength adaptations between autoregulated and standardized load prescription. Although non-significant, the sub-analysis demonstrated a small effect (SMD = 0.28) for greater 1RM strength when autoregulated load prescription employed a significantly higher relative intensity compared to standardized load prescription. It has been consistently supported that 1RM strength adaptations are primarily driven by high relative intensity training [50, 51], which agrees with the well documented force–velocity relationship (strength-speed continuum) [52, 53]. To illustrate, Graham and Cleather [20] demonstrated that when volume load, sets, and repetitions per set were matched, autoregulating load using RIR-based RPE to utilize a significantly higher relative intensity (percentage of 1RM) than the standardized PBT group resulted in significantly greater 1RM strength in both the back squat and front squat. Therefore, it appears that autoregulated load prescription has the potential to be slightly superior to standardized load prescription for 1RM strength if it enables a greater load (percentage of 1RM) to be utilized while accounting for an individual’s fluctuations and changes in 1RM.

The pooled analysis revealed near-significance (*p* = 0.06) with a small effect (SMD = 0.30) favoring subjective autoregulated over standardized load prescription; however, objective autoregulated load prescription failed to reveal any differences (*p* = 0.62; SMD = 0.10). The underlying rationale for these results is plausibly due to the objective autoregulated load prescription groups training at similar relative intensities as the standardized load prescription groups in all three of the objective load autoregulation studies [15, 19, 22]. In contrast, subjective autoregulated load prescription employed a significantly higher relative intensity than standardized load prescription for 50% of the total exercises in the subjective load autoregulation studies [20, 21, 23]. Moreover, an important consideration is that the studies employing subjective load autoregulation [20, 21, 23] were notably longer in duration than the studies employing objective load autoregulation [15, 19, 22]. Therefore, the reason for the marginally greater 1RM strength adaptations observed with subjective load autoregulation is likely twofold: (1) enabling a higher average RPE to be achieved; thus, resulting in a higher relative intensity being employed; and (2) longer training interventions; thus, providing a longer duration for strength adaptations to ensue. Interestingly, the results from this meta-analysis are conflicting with the sole study to date to directly compare subjective versus objective load autoregulation: a 12-week randomized cross-over design by Shattock and Tee [16] revealed that the 1RM strength improvements for objective load autoregulation (VBT) were significantly greater than subjective load autoregulation (RPE) in both the back squat (*p* = 0.00001; ES: 1.37) and bench press (*p* = 0.003; ES: 0.98). As the numbers of sets and repetitions per set were matched between groups, the significantly greater 1RM strength improvements following objective load autoregulation were postulated to be due to training at a higher average RPE (higher relative intensity) than the subjective load autoregulation protocol since it has been supported that subjective intra-set RPE ratings tend to be overestimated [11]. Furthermore, evidence has supported that velocity feedback improves competitiveness and motivation to train [54]; ensuring maximal intended concentric velocity, which results in significantly greater 1RM strength compared to half-maximal intended concentric velocity training [55].

Finally, no noteworthy differences in 1RM strength were demonstrated between autoregulated and standardized load prescription when sub-analyses on training frequency, volume differences, exercise type, and additional exercises were conducted. Based on the meta-analytic findings from the present review it appears that autoregulated and standardized load prescription are similarly effective at eliciting considerable improvements in 1RM strength adaptations. These findings may be attributable to the limitations and lack of optimization in the presently employed autoregulatory strategies.

#### Primary Limitations

RPE-based training often autoregulates load prescription by attempting to standardize the inter-individual proximity to failure [20, 21, 23]; however, it contains a limitation: subjectivity [7, 11]. To illustrate, when participants performed a set to failure at 70% of 1RM in the back squat and verbally indicated when they believed that they were at a 5, 7, and 9 RPE, their actual RPE values were ~ 5, 4, and 2 repetitions below the predicted RPE, respectively [11]. Despite the limitations of RPE-based training, the ability to accurately predict RPE improves from set-to-set and when performed closer to failure [7]; therefore, it may serve as a suitable autoregulatory strategy in certain settings (i.e., clinical).

Banyard et al. [22] and Orange et al. [19] employed individualized load-velocity profiles to prescribe and autoregulate load from set-to-set. Average concentric velocity (ACV) is reliable from session-to-session in the back squat at 20–90% of 1RM [56]; therefore, prescribing an individualized first repetition average concentric velocity (FRV) corresponding to a particular percentage of 1RM from an individualized load-velocity profile is a feasible strategy to dictate and autoregulate relative training load. Specifically, in the study conducted by Banyard et al. [22], if the ACV of all repetitions within a set was ± 0.06 m per second (m^.^s^−1^) outside the target ACV corresponding to a particular percentage of 1RM, the load was adjusted by a universal ± 5% of 1RM. However, ACV decreases linearly from repetition-to-repetition as one approaches failure [25]; Morán-Navarro et al. [13] reported that changes in ACV of 0.03 m^.^s^−1^ can produce a difference of two RPE in the back squat. As a result, load was seemingly reduced prematurely as evidenced by the VBT group training at similar relative intensities as the PBT group (~ 69 and ~ 71% of 1RM, respectively) [22]. Although a universal load adjustment of ± 5% of 1RM was still prescribed in Orange et al. [19], the load was appropriately adjusted from the FRV (rather than the ACV of all repetitions). Despite this, the intervention simply did not enable adequate progressive overload to observe any significant differences in 1RM strength improvements between the VBT and PBT groups [19]. In one of the most recent load autoregulation studies, individualized set-to-set load adjustments based off the actual percentage of 1RM from the previous set were performed; however, no differences in 1RM strength were reported between individualized and group load-velocity profiles following the 6-week training protocol [18].

An additional velocity-based load autoregulation strategy is the use of ACV zones [15, 16]. Although ACV zones account for specific zones on the force–velocity continuum [52, 53], they can correspond to considerably large ranges of relative intensities and proximities to failure. For example, if 3 sets of 8 repetitions in an ACV zone of 0.60–0.40 m^.^s^−1^ are prescribed, some individuals may commence the set at 0.60 m^.^s^−1^ and terminate the set at 0.45 m^.^s^−1^, while other individuals may commence the set at 0.50 m^.^s^−1^ and terminate the set at 0.40 m^.^s^−1^. However, Morán-Navarro et al. [13] reported group ACVs of ~ 0.49, 0.45, and 0.40 m^.^s^−1^ at a 4, 6, and 8 RPE, respectively during a single set to failure in the back squat. Therefore, terminating the set at an ACV of ~ 0.45 and 0.40 m^.^s^−1^ in the back squat is associated with ~ 6 and 8 RPE, respectively [13]; thus, ACV zones also fail to equate for proximity to failure and intra-set neuromuscular fatigue.

Finally, 1RM prediction from individualized regression equations of submaximal ACV may be inaccurate for determining sessional 1RM to prescribe load and autoregulate volume within a training session, or for tracking estimated 1RM to monitor chronic strength improvements across a training intervention [57–59]. In theory, the efficacy of 1RM prediction from individualized regression equations of submaximal ACV is predicated upon its ability to accurately predict 1RM, which has lacked consistent support [60–68]. These 1RM prediction methods have been purported to accurately predict the actual 1RM for most machine-based exercises [67, 68]; however, they over-predict the actual 1RM for barbell exercises in all [60–63, 65] but two [64, 66] studies. To summarize, the present load autoregulation strategies likely require further optimization encompassing individualized load adjustments and accurate quantifications of proximity to failure.

### Volume Autoregulation

#### Muscular Strength

Velocity loss thresholds ≤ 25% were superior for increasing 1RM strength over velocity loss thresholds > 25%, which is plausibly attributable to more favorable neuromuscular adaptations [31, 32] and lower neuromuscular fatigue [25, 44]. To better contextualize the overall meta-analytic findings for neuromuscular adaptations contributing to 1RM strength, a recent study conducted by Pareja-Blanco et al. [32] comparing 0, 15, 25, and 50% velocity loss demonstrated that 25% velocity loss resulted in the greatest 1RM strength improvements and was the velocity loss threshold that significantly increased late rate of force development. Crucially, maximizing late rate of force development is the paramount component of the force–time curve to optimize 1RM strength as shifting the force–time curve towards late rate of force development-oriented profiles optimizes the peak absolute force (absolute load) that can be produced [69, 70]. It is important to recognize that when percentage of 1RM and total repetitions are matched, maximizing intra-set force production with alternative set structures does not result in meaningfully different chronic strength adaptations compared to traditional sets as supported in two recent systematic reviews and meta-analyses [26, 37]. Rather, increases in the force component of the force–velocity curve for 1RM strength adaptations are largely contingent upon the load (i.e., percentage of 1RM) employed [50, 51, 71, 72]. Our meta-analytic data suggest that there may be a non-significant dose–response relationship between autoregulated velocity loss and 1RM strength adaptations from zero to ~ 25% velocity loss when training up to ~ 85% of 1RM possibly due to adaptation shifts from early to late rate of force development-oriented profiles [31, 32]. Moreover, velocity loss thresholds exceeding 25% typically result in meaningfully lower 1RM strength adaptations caused by unfavorable neuromuscular adaptations concomitant with counterproductive neuromuscular fatigue.

Velocity loss thresholds ≤ 25% were superior to velocity loss thresholds > 25% for increasing strength when the training intervention was 8 weeks in length, but not when it was < 8 weeks. This supports the contention that the advantages of lower intra-set fatigue for strength development are predominantly a result of chronic neuromuscular adaptations. A recent investigation by Pareja-Blanco et al. [31] demonstrated that 0 and 10% velocity loss significantly decreased vastus medialis muscle displacement, which is an indication of increased muscle stiffness [73, 74]; a property that mediates the force capabilities of skeletal muscle’s contractile elements [75]. Training at 0% velocity loss significantly increased early rate of force development [32], while 40% velocity loss significantly decreased early rate of force development [31]. Moreover, 40% velocity loss significantly increases vastus lateralis delay time [31], which has a positive relationship with myosin heavy chain I adaptations [76]. Rate of force development is a vital component for overcoming the sticking point in compound barbell lifts [77] and the decrease in rate of force development correlates with a decrease in the percentage of type IIX fibers [78, 79]. Importantly, type IIX fibers possess greater cross-bridge cycling rates compared to type I fibers [80] and are essential for enhancing muscle fiber conduction velocity [81]. Although 15% velocity loss elicits high peak muscle excitation [32], which is related to increased neural drive, rate coding, and sarcoplasmic reticulum calcium kinetics [82–85], training with 15% velocity loss does not change early or late rate of force development [32].

Compared to training with > 25% velocity loss, training with 25, 20, and 10% velocity loss all revealed small effects for increasing 1RM; however, both 15 and 0% velocity loss failed to reveal greater benefits for 1RM strength adaptations. For optimizing 1RM strength, it appears that the velocity loss thresholds may be polarized at 10% and 20–25% up to ~ 85% of 1RM as a viable strategy to minimize neuromuscular fatigue and maximize late rate of force development, respectively [28, 32]. However, upon further inspection of the included studies, it must be noted that the investigation conducted by Held et al. [28] comparing velocity loss thresholds of 10% at ~ 8 RPE to- repetition-failure training at 80% of 1RM in the barbell back squat, bench press, deadlift, bench row, and power clean also involved a high volume of additional exercises every day apart from the resistance training protocol (i.e., 90-min and 120-min of low-intensity rowing and cycling, respectively, on day 6 of the 7-day microcycle). Therefore, due to the possibility that the concurrent endurance training in Held et al. [28] confounded the findings, the sub-analysis comparing a velocity loss threshold of 10% to > 25% with Held et al. [28] excluded failed to reveal a small effect (MD = 1.65, *p* = 0.32, SMD = 0.16). As 20–25% velocity loss provided the greatest effect (SMD = 0.30), it may be suggested that 0–25% velocity loss with most training allocated at ~ 20–25% (the highest threshold compared to training at ~ 9.5 RPE—failure) is recommended to optimize 1RM strength.

Our pooled analysis revealed that velocity loss thresholds ≤ 25% resulted in significantly greater 1RM strength adaptations than velocity loss thresholds > 25% when additional exercise (beyond resistance training) was performed; however, no differences were observed when no additional exercise was performed. Therefore, it appears that the utility of velocity loss thresholds ≤ 25% for increasing 1RM strength may be most apparent when additional exercise beyond the resistance training protocol is performed (i.e., in-season) to minimize neuromuscular fatigue [28, 44]. Although relative intensity- and relative volume-equated failure training elevates muscle damage and elongates recovery time considerably compared to non-failure training [40], high volume and high velocity loss training results in greater neuromuscular fatigue and delayed recovery compared to high intensity and high RPE training [44, 86, 87]. For example, the counter-movement jump required 48-h to recover following 3 sets at 60% of 1RM with 40% velocity loss (~ 5.5 RPE) in the smith machine back squat; however, it was recovered within 6-h following 3 sets at 80% of 1RM with 20% velocity loss (~ 7 RPE) [86]. Pareja-Blanco et al. [44] also revealed that when set volume and velocity loss was equated (~ 17–23% velocity loss), the counter-movement jump was recovered at 24-h post-intervention regardless of training at a 4, 5, 6, 7, or 8 RPE at 70, 75, 80, 85, and 90% of 1RM in the smith machine back squat, respectively. Notably, Watkins and colleagues [88] investigated the counter-movement jump as an indicator of neuromuscular fatigue and readiness [89]; demonstrating that ~ 8% decrease in counter-movement jump height resulted in a ~ 28% decrease in the number of repetitions performed at 80% of 1RM in the back squat. Upon synthesis of the available evidence, it appears that training at velocity loss thresholds of 0–25% may limit undesirable neuromuscular fatigue; thereby, enabling the utilization of higher percentages of 1RM more frequently to train the high-force component of the force–velocity profile for 1RM strength adaptations.

#### Muscular Hypertrophy

Training with velocity loss thresholds > 25% resulted in significantly greater muscle hypertrophy than velocity loss thresholds ≤ 25%. This finding is consistent with the literature corroborating that there is a malleable inverted U-curve of optimal training volume for strength [90] and hypertrophy [91–93] that is individual-specific [94]. It is important to recognize that although the velocity loss threshold groups within each study were equated for set volume, velocity loss thresholds > 25% were associated with substantially greater total relative volume [30–32]. However, two recent systematic reviews and meta-analyses reported no difference in hypertrophy between traditional sets (higher intra-set fatigue) and alternative set structures (lower intra-set fatigue) when total relative volume was equated [26, 37]. Therefore, it appears that the significantly greater hypertrophy observed at > 25% velocity loss in our meta-analysis could be attributable to the greater total relative volume accumulated rather than due to the magnitude of intra-set fatigue (velocity loss threshold) achieved.

Although considerable intra-set fatigue and metabolic stress is unnecessary to stimulate hypertrophy [95], our meta-analysis suggests that a minimal velocity loss threshold of ~ 20–25% may be required to optimize hypertrophy as exemplified by small-to-moderate effects in favor of > 25% velocity loss arising in all sub-analyses including velocity loss thresholds at or below 20%. Despite this, future studies equated for relative volume that report intra-set fatigue with velocity loss and proximity to failure with a precise strategy are required to support or refute this finding and to establish the specific inter-dependent training status, velocity loss, and proximity to failure thresholds to optimize hypertrophy. Upon further inspection of the individual studies included in this meta-analysis, Pareja-Blanco et al. [31] demonstrated that despite no significant difference between 10 and 20% velocity loss in relative volume, number of sets, number of repetitions performed within the set, and percentage of 1RM, and despite training at nearly identical proximities to failure for the entire 8-week intervention (10% velocity loss: ~ 4–7.5 RPE; 20% velocity loss: ~ 4.5–8 RPE), 20% velocity loss elicited more than threefold greater hypertrophy compared to 10% velocity loss. Higher velocity loss thresholds result in elevated metabolite accumulation [25, 96], which has been postulated to amplify the hypertrophic response that is primarily induced from mechanical tension [97]. Consequently, it may be hypothesized that velocity loss thresholds < ~ 20% are inadequate at accumulating sufficient metabolites (i.e., lactate) to assist in augmenting optimal hypertrophic responses [97–99]; however, the evidence that lactate modulates anabolic signaling during resistance training in humans is conflicting [100, 101]. It is not entirely clear whether the results of this meta-analysis are due to differences between velocity loss threshold groups for relative volume, intra-set fatigue, or a combination of both.

Velocity loss thresholds of ≤ ~ 25% and > ~ 35% are associated with the preservation and reduction, respectively, of muscle fiber phenotypic characteristics that are favorable for enhancing 1RM strength adaptations [30, 102]. For example, when training at 70–85% of 1RM, 20% velocity loss at ~ 5–8 RPE preserved myosin heavy chain IIX muscle fiber percentage, whereas 40% velocity loss at ~ 9.5 RPE – failure reduced the type IIX fiber pool (i.e., with a conversion to slower fiber types) [30]. This is consistent with the electromyography literature and implies that high-threshold motor units of prime movers are activated initially within a set at ≥ ~ 70% of 1RM in trained individuals [30, 103–105]. However, 40% velocity loss increased vastus lateralis and vastus intermedius muscle volume to a significantly greater magnitude, suggesting that motor unit activation of synergists increases as velocity loss increases above ~ 25–35% [30]. It may be argued that hypertrophy should be related to increases in strength as muscle size accounts for ≥ 70% of the variance in strength in trained individuals [106–111]. Collectively, it may be suggested that if the primary goal is to increase strength, the majority of training should be performed at ~ 20–25% velocity loss to ensure and optimize hypertrophy while preserving type II muscle fiber phenotypic characteristics. Conversely, if the goal is to increase strength along with hypertrophy, velocity loss thresholds exceeding 25% may be employed; however, performing training at ~ 20–25% velocity loss for compound exercises with accessory exercises that target the synergist musculature may also accomplish this goal. Nonetheless, if increasing strength is of minimal importance, allocating the majority of training at a minimum of ~ 20–25% velocity loss may be recommended to optimize hypertrophy. A periodized approach integrating higher velocity loss initially within a macrocycle and decreasing towards lower velocity loss prior to competition may be employed in athletic programming settings to promote the spectrum of hypertrophy and strength velocity loss-specific adaptations in a sequential order for maximal performance.

#### Primary Limitations

Although velocity loss can accurately quantify acute intra-set neuromuscular and metabolic fatigue [25], it cannot precisely quantify proximity to failure [112, 113]. For example, in one of the preliminary longitudinal velocity loss studies by Pareja-Blanco et al. [31], a prescription would have been 3 sets at 70% of 1RM with a 20% velocity loss; stipulating that the FRV on each set corresponded to 70% of the individual’s 1RM. However, for example, one individual may have had an FRV of 0.60 m^.^s^−1^ at 70% of 1RM; thus, a 20% velocity loss would have terminated the set at an ACV of 0.48 m^.^s^−1^, which is associated with ~ 4 RPE [13]. Conversely, another individual may have had an FRV of 0.50 m^.^s^−1^ at 70% of 1RM; thus, a 20% velocity loss would have terminated the set at an ACV of 0.40 m^.^s^−1^, which is associated with ~ 8 RPE [13]. To further illustrate, a separate investigation by the same researchers reported that the 40% velocity loss group performed ~ 56% of the sets to failure, indicating that 40% velocity loss corresponded to differing intra- and inter-individual proximities to failure [30].

Most importantly, velocity loss is primarily influenced by the number of repetitions performed within the set rather than the proximity to failure (i.e., low velocity loss is not necessarily indicative of low RPE) [25]. For example, if an individual has an ACV corresponding to an 8 RPE of 0.30 m^.^s^−1^ and a velocity decay (change in velocity per change in RPE) of 0.04 m^.^s^−1^, the velocity loss for 3 and 7 repetitions at an 8 RPE would equate to 21 and 44%, respectively. Moreover, each velocity loss threshold is load- and lift-specific; corresponding to a different proximity to failure depending on the percentage of 1RM and exercise employed [112–114]. Furthermore, the inter-individual range in repetitions reported in the back squat at 10, 20, and 30% velocity loss with an FRV of 0.70 m^.^s^−1^ was 2–11, 4–19, and 4–24 repetitions, respectively [115]. Therefore, due to the extensive range in repetitions that can be performed at each percentage of 1RM [7, 8] and at each velocity loss threshold [115], as well as the FRV [116], the ACV corresponding to a specific RPE [13], and the individualized velocity decay, each individual will experience varying degrees of fatigue, terminate each set at a different proximity to failure, and perform a different number of repetitions and magnitude of relative volume. Conclusively, an autoregulatory model conceptualizing the overarching results from this meta-analysis may be required to suggest avenues for future research and potential practical applications.

### Future Directions and Practical Applications

Although the results from this meta-analysis demonstrated significant differences between velocity loss thresholds ≤ 25% and > 25% for both strength and hypertrophy adaptations, several limitations require further investigation. Future studies should compare different velocity loss thresholds and equate for total relative volume and relative intensity to detect whether the specific adaptations are due to differences in the amount of volume performed, magnitude of intra-set fatigue, or a combination of both. Additionally, future research is also warranted to investigate the chronic effects of load and volume autoregulation in clinical settings when standardized resistance training strategies may be contraindicated [117]. Due to the limitations in which velocity-based load and volume autoregulation strategies have been employed and considering the conflicting findings between this meta-analysis and the sole study to date to directly compare objective to subjective load autoregulation [16], a separate autoregulatory model potentially warrants conceptualization and investigation within autoregulatory contexts. Accordingly, a model integrating individualized last repetition average concentric velocity (LRV) may be a potential autoregulatory model to conceptualize the individual- and lift-specific relationship between LRV, repetitions performed, percentage of 1RM, proximity to failure (RPE), and intra-set fatigue (velocity loss) based on emerging evidence [13, 118, 119].

In practical settings, velocity loss zones from our meta-analysis may be conceptualized contingent upon the primary goal of the individual or training phase within a theoretical model; however, LRVs may be utilized as a prescription strategy within the velocity loss zones to quantify proximity to failure and potentially help rectify the limitations of the present mutually exclusive autoregulatory prescription strategies. From a more holistic programming perspective, a periodized approach whereby higher velocity loss with higher relative volume and lower RPE change over time towards lower velocity loss with higher relative intensity and higher RPE may be recommended. An integrated approach may be a plausible advantageous strategy for optimizing total hypertrophy to potentiate neuromuscular adaptations and limit neuromuscular fatigue for peak performance at the time of primary importance [120]. Perhaps most noteworthy, the efficacy of LRVs and potential model warrants future investigation in: (1) a longitudinal volume autoregulation study comparing LRV stops to RPE stops and velocity loss thresholds; and (2) a longitudinal load autoregulation study comparing LRV-based training to RPE-based training and individualized PBT.

## Conclusion

The results of this systematic review and meta-analysis provide novel evidence regarding the effect of autoregulated load and volume prescription on muscular strength and hypertrophy adaptations. Specifically, autoregulated and standardized load prescription demonstrated similar improvements in 1RM strength. Low-moderate velocity loss thresholds of 0–25% (i.e., lower intra-set fatigue) and moderate-high velocity loss thresholds of > 20–25% (i.e., higher intra-set fatigue) produce the greatest improvements in 1RM strength and muscle hypertrophy, respectively. Velocity loss thresholds of ~ 20–25% may optimize 1RM strength adaptations by maximizing favorable chronic hypertrophy adaptations (i.e., type II phenotypic characteristics), chronic neuromuscular adaptations (i.e., late rate of force development), while minimizing unnecessary acute neuromuscular fatigue. Collectively, integrating load and volume autoregulation is a plausible strategy to aid in the systematic individualization of resistance training programming prescriptions, acute responses, chronic adaptations, and performance outcomes.

## Supplementary Information

**Additional file 1: Table S1.** Methodological quality of included studies on load autoregulation.

**Additional file 2: Table S2.** Methodological quality of included studies on volume autoregulation.

**Additional file 3: Fig. S1.** Funnel plot for fixed effects meta-analysis of the mean differences in one-repetition maximum strength adaptations comparing autoregulated to standardized load prescription with subgroup analysis comparing subjective to objective autoregulation. *MD* mean difference, *SE* standard error.

**Additional file 4: Fig. S2.** Funnel plot for fixed effects meta-analysis of the mean differences in one-repetition maximum strength adaptations comparing ≤ 25% to > 25% velocity loss with subgroup analysis comparing additional to no additional exercise apart from the main comparator resistance training protocol. *MD* mean difference, *SE* standard error.

**Additional file 5: Fig. S3.** Funnel plot for fixed effects meta-analysis of the mean differences in cross-sectional area hypertrophy adaptations comparing > 25% to ≤ 25% velocity loss. *MD* mean difference, *SE* standard error.

**Additional file 6: Table S3.** Results from sub-analyses for 1RM strength between autoregulated and standardized load prescription.

**Additional file 7: Table S4.** Results from sub-analyses for 1RM strength between ≤ 25% velocity loss and > 25% velocity loss.

**Additional file 8: Table S5.** Results from sub-analyses for 1RM strength between respective velocity loss and > 25% velocity loss.

**Additional file 9: Table S6.** Results from sub-analyses for CSA hypertrophy between > 25% velocity loss and respective velocity loss.

## Data Availability

The datasets generated and/or analysed during the current review are available from the corresponding author on reasonable request.

## Abbreviations

ACV: Average concentric velocity
CI: Confidence interval
CSA: Cross-sectional area
ES: Effect size
FRV: Individualized first repetition average concentric velocity
LRV: Individualized last repetition average concentric velocity
MD: Mean difference
m^.^s^−1^: Metres per second
PBT: Percentage-based training
PICOS: Participants, interventions, comparisons, outcomes, and study design
PRISMA: Preferred Reporting Items for Systematic Reviews and Meta-Analyses
PROSPERO: International Prospective Register of Systematic Reviews
RIR-based RPE scale: Repetitions in reserve-based rating of perceived exertion scale
RoB 2: Revised Cochrane risk-of-bias tool for randomized trials
RT: Resistance training
SD: Standard deviation
SMD: Standardized mean difference
VBT: Velocity-based training
1RM: One-repetition maximum
