# Supplementary material for: The Effect of Load and Volume Autoregulation on Muscular Strength and Hypertrophy: A Systematic Review and Meta-Analysis
Source: Sports Med Open. 2022 Jan 15;8:9. doi: 10.1186/s40798-021-00404-9 (PMC8762534; doi:10.1186/s40798-021-00404-9)
Supplement: Supplementary file 1 — Additional file 1: Table S1. Methodological quality of included studies on load autoregulation. [file 40798_2021_404_MOESM1_ESM.pdf]

Electronic Supplementary Table S1 Cover Page

Article title: The Effect of Load and Volume Autoregulation on Muscular Strength and Hypertrophy: A Systematic Review and Meta-Analysis

Journal name: Sports Medicine - Open

Author names: Landyn M. Hickmott[1], Philip D. Chilibeck[2], Keely A. Shaw[2], Scotty J. Butcher[3]

Author affiliations:

College of Medicine, Health Sciences Program, University of Saskatchewan, Saskatoon, Canada[1]

College of Kinesiology, University of Saskatchewan, Saskatoon, Canada[2]

School of Rehabilitation Science, University of Saskatchewan, Saskatoon, Canada[3]

Corresponding author: Landyn M. Hickmott, lmh896@usask.ca

Electronic Supplementary Table S1 Methodological quality of included studies on load autoregulation

Study	Random sequence generation (selection bias)	Allocation concealment (selection bias)	Blinding of participants and researchers (performance bias)	Blinding of outcome assessment (detection bias)	Incomplete outcome data (attrition bias)	Selective reporting (reporting bias)	Overall bias
Arede et al. [23]	Some	Low	Some	Low	Some	Some	Some
Banyard et al. [22]	Some	Low	Low	Low	Some	Some	Some
Dorrell et al. [15]	Some	Low	Some	Low	Some	Some	Some
Dorrell et al. [18]	Some	Low	Some	Low	Some	Some	Some
Graham and Cleather [20]	Some	Some	Some	Low	Some	Some	Some
Helms et al. [21]	Some	Low	High	Low	Some	Some	Some
Orange et al. [19]	Some	Low	Low	Low	Some	Some	Some
Shattock and Tee [16]	Some	Low	Some	Low	Some	Some	Some
