# Supplementary material for: The Effect of Load and Volume Autoregulation on Muscular Strength and Hypertrophy: A Systematic Review and Meta-Analysis
Source: Sports Med Open. 2022 Jan 15;8:9. doi: 10.1186/s40798-021-00404-9 (PMC8762534; doi:10.1186/s40798-021-00404-9)
Supplement: Supplementary file 2 — Additional file 2: Table S2. Methodological quality of included studies on volume autoregulation. [file 40798_2021_404_MOESM2_ESM.pdf]

Electronic Supplementary Table S2 Cover Page

Article title: The Effect of Load and Volume Autoregulation on Muscular Strength and Hypertrophy: A Systematic Review and Meta-Analysis

Journal name: Sports Medicine - Open

Author names: Landyn M. Hickmott[1], Philip D. Chilibeck[2], Keely A. Shaw[2], Scotty J. Butcher[3]

Author affiliations:

College of Medicine, Health Sciences Program, University of Saskatchewan, Saskatoon, Canada[1]

College of Kinesiology, University of Saskatchewan, Saskatoon, Canada[2]

School of Rehabilitation Science, University of Saskatchewan, Saskatoon, Canada[3]

Corresponding author: Landyn M. Hickmott, lmh896@usask.ca

Electronic Supplementary Table S2 Methodological quality of included studies on volume autoregulation

Study	Random sequence generation (selection bias)	Allocation concealment (selection bias)	Blinding of participants and researchers (performance bias)	Blinding of outcome assessment (detection bias)	Incomplete outcome data (attrition bias)	Selective reporting (reporting bias)	Overall bias
Galiano et al. [27]	Some	Low	Low	Low	Some	Some	Some
Held et al. [28]	Some	Low	Low	Low	Some	Some	Some
Pareja-Blanco et al. [29]	Some	Low	Low	Low	Some	Some	Some
Pareja-Blanco et al. [30]	Some	Low	Low	Low	Some	Some	Some
Pareja-Blanco et al. [31]	Some	Low	Low	Low	Some	Some	Some
Pareja-Blanco et al. [32]	Some	Low	Low	Low	Some	Some	Some
Rodiles-Guerrero et al. [33]	High	Low	Some	Low	Some	Some	Some
Rodríguez-Rosell et al. [34]	Some	Low	Low	Low	Some	Some	Some
Rodríguez-Rosell et al. [35]	Some	Low	Low	Low	Some	Some	Some
Sánchez-Moreno et al. [36]	Some	Low	Low	Low	Some	Some	Some
