# Supplementary material for: The Effect of Load and Volume Autoregulation on Muscular Strength and Hypertrophy: A Systematic Review and Meta-Analysis
Source: Sports Med Open. 2022 Jan 15;8:9. doi: 10.1186/s40798-021-00404-9 (PMC8762534; doi:10.1186/s40798-021-00404-9)
Supplement: Supplementary file 5 — Additional file 5: Fig. S3. Funnel plot for fixed effects meta-analysis of the mean differences in cross-sectional area hypertrophy adaptations comparing > 25% to ≤ 25% velocity loss. MD mean difference, SE standard error. [file 40798_2021_404_MOESM5_ESM.pdf]

Electronic Supplementary Figure S3 Cover Page

Article title: The Effect of Load and Volume Autoregulation on Muscular Strength and Hypertrophy: A Systematic Review and Meta-Analysis

Journal name: Sports Medicine - Open

Author names: Landyn M. Hickmott[1], Philip D. Chilibeck[2], Keely A. Shaw[2], Scotty J. Butcher[3]

Author affiliations:

College of Medicine, Health Sciences Program, University of Saskatchewan, Saskatoon, Canada[1]

College of Kinesiology, University of Saskatchewan, Saskatoon, Canada[2]

School of Rehabilitation Science, University of Saskatchewan, Saskatoon, Canada[3]

Corresponding author: Landyn M. Hickmott, lmh896@usask.ca

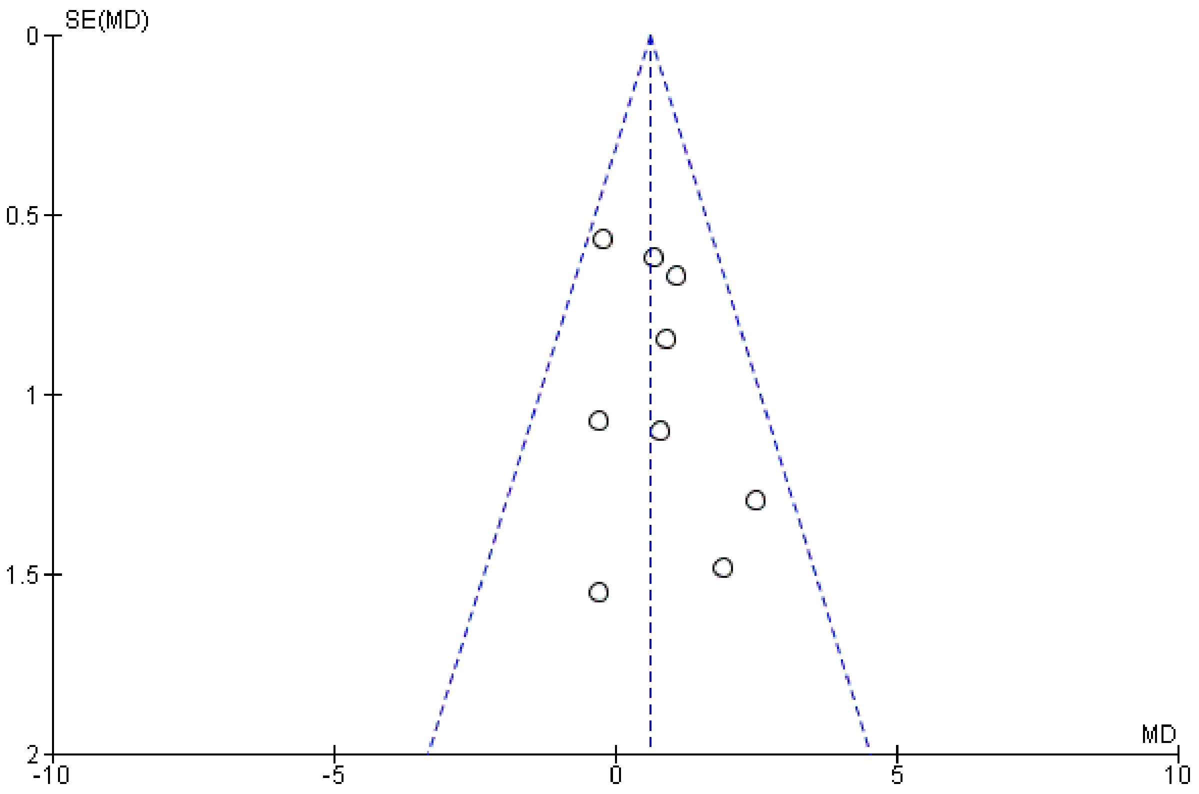

Electronic Supplementary Figure S3 Funnel plot for fixed effects meta-analysis of the mean differences in cross-sectional area hypertrophy adaptations comparing >25% to ≤25% velocity loss. *MD* mean difference, *SE* standard error
