# Supplementary material for: The Effect of Load and Volume Autoregulation on Muscular Strength and Hypertrophy: A Systematic Review and Meta-Analysis
Source: Sports Med Open. 2022 Jan 15;8:9. doi: 10.1186/s40798-021-00404-9 (PMC8762534; doi:10.1186/s40798-021-00404-9)
Supplement: Supplementary file 6 — Additional file 6: Table S3. Results from sub-analyses for 1RM strength between autoregulated and standardized load prescription. [file 40798_2021_404_MOESM6_ESM.pdf]

Electronic Supplementary Table S3 Cover Page

Article title: The Effect of Load and Volume Autoregulation on Muscular Strength and Hypertrophy: A Systematic Review and Meta-Analysis

Journal name: Sports Medicine - Open

Author names: Landyn M. Hickmott[1], Philip D. Chilibeck[2], Keely A. Shaw[2], Scotty J. Butcher[3]

Author affiliations:

College of Medicine, Health Sciences Program, University of Saskatchewan, Saskatoon, Canada[1]

College of Kinesiology, University of Saskatchewan, Saskatoon, Canada[2]

School of Rehabilitation Science, University of Saskatchewan, Saskatoon, Canada[3]

Corresponding author: Landyn M. Hickmott, lmh896@usask.ca

Electronic Supplementary Table S3 Results from sub-analyses for 1RM strength between autoregulated and standardized load prescription

Sub-analysis	Test of effect and variability				Heterogeneity				Test for subgroup differences
Type	MD (kg)	95% CI (kg)	p	SMD	I^2 (%)	Chi^2 (Q-Value)	df	p	p
≥8 weeks intervention length	3.15	-0.14 to 6.45	0.06	0.30	0.00	0.55	5	0.99	0.35
<8 weeks intervention length	0.88	-2.59 to 4.34	0.62	0.10	0.00	0.95	5	0.97	
3 times per week frequency	0.98	-4.70 to 6.65	0.74	0.09	0.00	0.17	2	0.92	0.68
<3 times per week frequency	2.31	-0.33 to 4.94	0.09	0.25	0.00	2.03	8	0.98	
Volume controlled	2.12	-2.06 to 6.31	0.32	0.19	0.00	1.78	5	0.88	0.98
Volume uncontrolled	2.05	-0.86 to 4.95	0.17	0.23	0.00	0.59	5	0.99	
Relative intensity significantly greater for autoregulated over standardized	3.85	-2.27 to 9.97	0.22	0.28	0.00	0.46	2	0.79	0.54
Relative intensity not significantly different between subgroups	1.75	-0.84 to 4.34	0.19	0.17	0.00	1.53	8	0.99	
Lower body exercises	2.14	-1.53 to 5.81	0.25	0.19	0.00	1.78	7	0.97	0.96
Upper body exercises	2.02	-1.13 to 5.16	0.21	0.26	0.00	0.59	3	0.90	
Squat	1.92	-1.84 to 5.69	0.32	0.18	0.00	1.55	6	0.96	0.75
Bench press	2.83	-1.11 to 6.77	0.16	0.31	0.00	0.14	2	0.93	
Exercises additional to resistance training protocol were performed	2.71	-0.61 to 6.03	0.11	0.27	0.00	1.29	4	0.86	0.59
Exercises additional to resistance training protocol were not performed	1.39	-2.04 to 4.82	0.43	0.16	0.00	0.79	6	0.99	

*Statistically significant difference ($p \leq 0.05$)

CI confidence interval, *df* degrees of freedom, *kg* kilograms, *MD* mean difference, *SMD* standardized mean difference, *1RM* one-repetition maximum
