# Supplementary material for: The Effect of Load and Volume Autoregulation on Muscular Strength and Hypertrophy: A Systematic Review and Meta-Analysis
Source: Sports Med Open. 2022 Jan 15;8:9. doi: 10.1186/s40798-021-00404-9 (PMC8762534; doi:10.1186/s40798-021-00404-9)
Supplement: Supplementary file 7 — Additional file 7: Table S4. Results from sub-analyses for 1RM strength between ≤ 25% velocity loss and > 25% velocity loss. [file 40798_2021_404_MOESM7_ESM.pdf]

Electronic Supplementary Table S4 Cover Page

Article title: The Effect of Load and Volume Autoregulation on Muscular Strength and Hypertrophy: A Systematic Review and Meta-Analysis

Journal name: Sports Medicine - Open

Author names: Landyn M. Hickmott[1], Philip D. Chilibeck[2], Keely A. Shaw[2], Scotty J. Butcher[3]

Author affiliations:

College of Medicine, Health Sciences Program, University of Saskatchewan, Saskatoon, Canada[1]

College of Kinesiology, University of Saskatchewan, Saskatoon, Canada[2]

School of Rehabilitation Science, University of Saskatchewan, Saskatoon, Canada[3]

Corresponding author: Landyn M. Hickmott, lmh896@usask.ca

Electronic Supplementary Table S4 Results from sub-analyses for 1RM strength between ≤25% velocity loss and >25% velocity loss

Sub-analysis	Test of effect and variability				Heterogeneity				Test for subgroup differences
Type	MD (kg)	95% CI (kg)	p	SMD	I^2 (%)	Chi^2 (Q-Value)	df	p	p
8 weeks intervention length	2.99	0.76 to 5.22	0.009	0.29	0.00	13.97	14	0.45	0.19
<8 weeks intervention length	-0.33	-4.73 to 4.08	0.88	-0.02	0.00	0.20	2	0.90	
3 times per week frequency	-0.33	-4.73 to 4.08	0.88	-0.02	0.00	0.20	2	0.90	0.19
<3 times per week frequency	2.99	0.76 to 5.22	0.009	0.29	0.00	13.97	14	0.45	
Lower body exercises	4.40	1.18 to 7.61	0.007	0.36	0.00	5.93	9	0.75	0.11
Upper body exercises	1.02	-1.51 to 3.56	0.43	0.11	5.00	7.36	7	0.39	
Smith machine back squat	3.42	0.07 to 6.76	0.05	0.27	0.00	1.60	7	0.98	0.05
Smith machine / weight stack bench press	-1.03	-4.04 to 1.97	0.50	-0.09	0.00	0.99	4	0.91	
Free-weight exercises	7.49	3.14 to 11.84	0.0007	0.65	0.00	2.70	4	0.61	0.009
Machine-based exercises	0.95	-1.29 to 3.19	0.41	0.10	0.00	6.35	12	0.90	

*Statistically significant difference (p ≤ 0.05)
CI confidence interval, *df* degrees of freedom, *kg* kilograms, *MD* mean difference, *SMD* standardized mean difference, *1RM* one-repetition maximum
