# Supplementary material for: The Effect of Load and Volume Autoregulation on Muscular Strength and Hypertrophy: A Systematic Review and Meta-Analysis
Source: Sports Med Open. 2022 Jan 15;8:9. doi: 10.1186/s40798-021-00404-9 (PMC8762534; doi:10.1186/s40798-021-00404-9)
Supplement: Supplementary file 8 — Additional file 8: Table S5. Results from sub-analyses for 1RM strength between respective velocity loss and > 25% velocity loss. [file 40798_2021_404_MOESM8_ESM.pdf]

Electronic Supplementary Table S5 Cover Page

Article title: The Effect of Load and Volume Autoregulation on Muscular Strength and Hypertrophy: A Systematic Review and Meta-Analysis

Journal name: Sports Medicine - Open

Author names: Landyn M. Hickmott[1], Philip D. Chilibeck[2], Keely A. Shaw[2], Scotty J. Butcher[3]

Author affiliations:

College of Medicine, Health Sciences Program, University of Saskatchewan, Saskatoon, Canada[1]

College of Kinesiology, University of Saskatchewan, Saskatoon, Canada[2]

School of Rehabilitation Science, University of Saskatchewan, Saskatoon, Canada[3]

Corresponding author: Landyn M. Hickmott, lmh896@usask.ca

Electronic Supplementary Table S5 Results from sub-analyses for 1RM strength between respective velocity loss and >25% velocity loss

Sub-analysis	Test of effect and variability				Heterogeneity			
Velocity loss threshold	MD (kg)	95% CI (kg)	p	SMD	I^2 (%)	Chi^2 (Q-Value)	df	p
25%	3.38	-1.93 to 8.70	0.21	0.31	0.00	0.42	1	0.52
20%	3.42	-3.08 to 9.93	0.30	0.28	0.00	0.15	1	0.70
15%	-0.50	-6.89 to 5.88	0.88	-0.02	0.00	0.24	1	0.62
10%	3.56	0.77 to 6.34	0.01	0.35	11.00	10.15	9	0.34
0%	-1.56	-6.56 to 3.45	0.54	-0.11	0.00	0.87	1	0.35
20 – 25%	3.40	-0.72 to 7.52	0.11	0.30	0.00	0.57	3	0.90
15 – 25%	2.25	-1.21 to 5.71	0.20	0.20	0.00	1.82	5	0.87
10 – 25%	3.04	0.87 to 5.21	0.006	0.29	0.00	12.30	15	0.66
15 – 20%	1.42	-3.13 to 5.98	0.54	0.13	0.00	1.11	3	0.78
10 – 20%	2.98	0.60 to 5.35	0.01	0.28	0.00	11.87	13	0.54
0 – 20%	2.14	-0.00 to 4.29	0.05	0.22	2.00	15.31	15	0.43
10 – 15%	2.91	0.36 to 5.46	0.03	0.28	6.00	11.69	11	0.39
0 – 15%	1.99	-0.29 to 4.26	0.09	0.21	13.00	14.99	13	0.31
0 – 10%	2.35	-0.08 to 4.78	0.06	0.25	22.00	14.08	11	0.23

*Statistically significant difference ($p \leq 0.05$)

CI confidence interval, *df* degrees of freedom, *kg* kilograms, *MD* mean difference, *SMD* standardized mean difference, *1RM* one-repetition maximum
