# Supplementary material for: The Effect of Load and Volume Autoregulation on Muscular Strength and Hypertrophy: A Systematic Review and Meta-Analysis
Source: Sports Med Open. 2022 Jan 15;8:9. doi: 10.1186/s40798-021-00404-9 (PMC8762534; doi:10.1186/s40798-021-00404-9)
Supplement: Supplementary file 9 — Additional file 9: Table S6. Results from sub-analyses for CSA hypertrophy between > 25% velocity loss and respective velocity loss. [file 40798_2021_404_MOESM9_ESM.pdf]

Electronic Supplementary Table S6 Cover Page

Article title: The Effect of Load and Volume Autoregulation on Muscular Strength and Hypertrophy: A Systematic Review and Meta-Analysis

Journal name: Sports Medicine - Open

Author names: Landyn M. Hickmott[1], Philip D. Chilibeck[2], Keely A. Shaw[2], Scotty J. Butcher[3]

Author affiliations:

College of Medicine, Health Sciences Program, University of Saskatchewan, Saskatoon, Canada[1]

College of Kinesiology, University of Saskatchewan, Saskatoon, Canada[2]

School of Rehabilitation Science, University of Saskatchewan, Saskatoon, Canada[3]

Corresponding author: Landyn M. Hickmott, lmh896@usask.ca

Electronic Supplementary Table S6 Results from sub-analyses for CSA hypertrophy between >25% velocity loss and respective velocity loss

Sub-analysis	Test of effect and variability				Heterogeneity			
Velocity loss threshold	MD (cm^2)	95% CI (cm^2)	p	SMD	I^2 (%)	Chi^2 (Q-Value)	df	p
20 – 25%	0.36	-0.29 to 1.00	0.28	0.13	0.00	3.03	4	0.55
15 – 25%	0.43	-0.20 to 1.06	0.18	0.20	0.00	4.07	5	0.54
10 – 25%	0.46	-0.15 to 1.06	0.14	0.21	0.00	4.17	6	0.65
20%	0.39	-0.28 to 1.05	0.25	0.20	0.00	2.84	3	0.42
15 – 20%	0.46	-0.18 to 1.11	0.16	0.26	0.00	3.84	4	0.43
10 – 20%	0.49	-0.13 to 1.11	0.12	0.26	0.00	3.92	5	0.56
10 – 15%	1.19	-0.54 to 2.92	0.18	0.36	0.00	0.36	1	0.55
0 – 20%	0.64	0.07 to 1.20	0.03	0.34	0.00	6.31	7	0.50
0 – 15%	1.31	0.22 to 2.39	0.02	0.44	0.00	1.45	3	0.69
0 – 10%	1.21	0.04 to 2.38	0.04	0.44	0.00	1.27	2	0.53
0%	1.38	-0.01 to 2.77	0.05	0.53	6.00	1.07	1	0.30

*Statistically significant difference ($p \leq 0.05$)

CI confidence interval, *cm* centimetres, *CSA* cross-sectional area, *df* degrees of freedom, *MD* mean difference, *SMD* standardized mean difference
